# Biochemical characterization of essential cell division proteins FtsX and FtsE that mediate peptidoglycan hydrolysis by PcsB in *Streptococcus pneumoniae*

**DOI:** 10.1002/mbo3.366

**Published:** 2016-05-10

**Authors:** Ruchika Bajaj, Kevin E. Bruce, Amy L. Davidson, Britta E. Rued, Cynthia V. Stauffacher, Malcolm E. Winkler

**Affiliations:** ^1^ Department of Biological SciencesPurdue UniversityWest LafayetteIndiana47907; ^2^ Department of BiologyIndiana University BloomingtonBloomingtonIndiana47405

**Keywords:** ABC transporter‐like proteins, bacterial cell division, FtsEX:PcsB complex, peptidoglycan (PG) hydrolases, pneumococcus

## Abstract

The FtsEX:PcsB complex forms a molecular machine that carries out peptidoglycan (PG) hydrolysis during normal cell division of the major respiratory pathogenic bacterium, *Streptococcus pneumoniae* (pneumococcus). FtsX is an integral membrane protein and FtsE is a cytoplasmic ATPase that together structurally resemble ABC transporters. Instead of transport, FtsEX transduces signals from the cell division apparatus to stimulate PG hydrolysis by PcsB, which interacts with extracellular domains of FtsX. Structural studies of PcsB and one extracellular domain of FtsX have recently appeared, but little is known about the biochemical properties of the FtsE ATPase or the intact FtsX transducer protein. We report here purifications and characterizations of tagged FtsX and FtsE proteins. Pneumococcal FtsX‐GFP‐His and FtsX‐His could be overexpressed in *Escherichia coli* without toxicity, and FtsE‐His remained soluble during purification. FtsX‐His dimerizes in detergent micelles and when reconstituted in phospholipid nanodiscs. FtsE‐His binds an ATP analog with an affinity comparable to that of ATPase subunits of ABC transporters, and FtsE‐His preparations have a low, detectable ATPase activity. However, attempts to detect complexes of purified FtsX‐His, FtsE‐His, and PcsB‐His or coexpressed tagged FtsX and FtsE were not successful with the constructs and conditions tested so far. In working with nanodiscs, we found that PcsB‐His has an affinity for charged phospholipids, mediated partly by interactions with its coiled‐coil domain. Together, these findings represent first steps toward reconstituting the FtsEX:PcsB complex biochemically and provide information that may be relevant to the assembly of the complex on the surface of pneumococcal cells.

## Introduction

Peptidoglycan (PG) biosynthesis requires the coordinated activities of the synthetic penicillin‐binding proteins (PBPs) and the remodeling PG hydrolases (reviewed in [Vollmer et al. 2008; Typas et al. 2012; Vollmer 2012; Egan and Vollmer 2013; Egan et al. 2015]). Loss or misregulation of the activities of the PBPs and division PG hydrolases is catastrophic to bacterial cell morphology and results in cell death or severely misshapened cells, often in chains (see [Potluri et al. 2010; Young 2010; Barendt et al. 2011; Massidda et al. 2013; Boersma et al. 2015]). A principle that has emerged from recent work is that division PG hydrolases are usually autoinhibited [see (Vollmer et al. 2008; Vollmer 2012; Yang et al. 2012; Bartual et al. 2014; Mavrici et al. 2014)] and require interactions with regulatory proteins to activate synchronized cleavage of the PG cell wall. These regulatory proteins, in turn, are coupled to the cell division apparatus that coordinates synthetic and hydrolytic activities.

**Figure 1:**
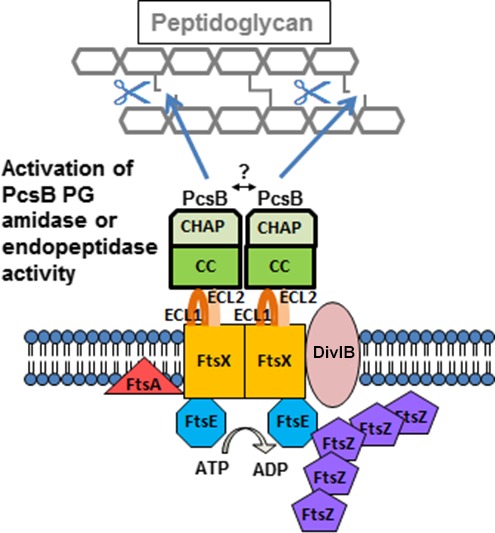
Model for regulated PG hydrolysis by the FtsEX:PcsB complex, whose function is essential in *S. pneumoniae*. The figure is modified from (Sham et al. 2011). The FtsEX:PcsB complex consists of the FtsE ATPase, the FtsX transmembrane protein, and the autoinhibited PcsB PG hydrolase. The FtsEX complex shares structural similarities to an ABC transporter, but does not transport a ligand. FtsX contains the large extracellular loop domain ECL1 and a small extracellular loop domain ECL2. Both extracellular loops are important in FtsEX:PcsB signal transduction. ECL1 interacts with the coiled‐coil (CC) domain of the PcsB PG hydrolase, which contains an autoinhibited CHAP catalytic domain. When activated, PcsB acts as a PG remodeling amidase or endopeptidase that cleaves PG peptides. At a specific point in cell division, the ATPase activity of FtsE is stimulated by interactions with midcell division proteins, such as FtsZ. The FtsE ATPase activity drives conformational changes in the transmembrane domain of FtsX, which also may interact with divisome proteins. The conformational change in the FtsX transmembrane domain is transmitted to the FtsX extracellular loops, ECL1 and ECL2, which in turn relieve the autoinhibition of the PcsB CHAP domain, allowing timed PG cleavage. The model is based on references (Arends et al. 2009; Sham et al. 2011, 2013; Mavrici et al. 2014; Yang et al. 2011, 2012; Meisner et al. 2013; Bartual et al. 2014; Mir et al. 2015). See text for additional details.

The FtsEX proteins were known to be involved in cell division (de Leeuw et al. 1999; Schmidt et al. 2004; Arends et al. 2009), and it was only recently shown that they function as regulators of division PG hydrolases (Sham et al. 2011, 2013; Yang et al. 2011; Mavrici et al. 2014). The general structure of FtsEX resembles an ABC transporter (Schmidt et al. 2004; Mir et al. 2006; Arends et al. 2009), but the function of FtsEX is to couple cell division to activation of bound PG hydrolases, which vary in different bacterial species (Sham et al. 2011; Yang et al. 2011; Meisner et al. 2013; Mavrici et al. 2014). In the ellipsoid‐shaped respiratory pathogen, *Streptococcus pneumoniae* (pneumococcus), FtsX interacts with the CHAP domain containing PG hydrolase, PcsB (Fig. 1) (Mesnage et al. 2008; Sham et al. 2011, 2013; Massidda et al. 2013; Bartual et al. 2014). FtsE, FtsX, and PcsB are essential for growth in some serotype strains of *S. pneumoniae* (Ng et al. 2003, 2004; Sham et al. 2013), and in other strains, their absence causes severely diminished growth and cell morphology defects (Giefing et al. 2008; Giefing‐Kroll et al. 2011). In strains where FtsEX:PcsB is essential, amino acid changes that inactivate the FtsE ATPase are not tolerated (Sham et al. 2013). A large extracellular loop domain of pneumococcal FtsX (ECL1^FtsX^) interacts with the coiled‐coil domain of PcsB (CC^PcsB^) (Sham et al. 2011), and both ECL1^FtsX^ and a small extracellular loop domain of FtsX (ECL2^FtsX^) transduce signals from the FtsE ATPase to activate PcsB PG hydrolase activity (Sham et al. 2013) (Fig. 1). Structural and physiological studies established that the CHAP domain of PcsB functions as a PG hydrolase that is autoinhibited either by folding of the CHAP domain into a cavity in the CC^PcsB^ domain of the same PcsB molecule or by domain swapping between dimers, wherein, the CC^PcsB^ domain of one monomer in the dimer inhibits the CHAP domain of the other monomer (Bartual et al. 2014).

Elements of the model for FtsEX:PcsB regulation (Fig. 1) apply to homologs from other bacteria, with some modifications. Similar to PcsB in *S. pneumoniae* (Fig. 1), the ECL1 domain of *B. subtilis* FtsX likely binds directly to and activates the CwlO PG hydrolase (Meisner et al. 2013). In contrast, the ECL1 domain of *E. coli* FtsX interacts with the EnvC adaptor protein that interacts with the autoinhibited AmiA/AmiB PG amidases (Yang et al. 2011, 2012; Peters et al. 2013). In *Mycobacterium tuberculosis*, PG hydrolase activity of RipC is stimulated by interactions between a coiled‐coil domain of RipC and the ECL1 domain of *M. tuberculosis* FtsX, which is called ECD (Mavrici et al. 2014). The ECD of *M. tuberculosis* FtsX forms a two‐lobed structure around a hydrophobic pocket that likely binds to the coiled‐coil domain of RipC during activation (Mavrici et al. 2014). Despite low amino acid sequence similarity, the overall distribution of secondary structure elements is similar in the structures of the ECL1^FtsX^ domains of *M. tuberculosis* and *S. pneumoniae*, with some differences (Mavrici et al. 2014; Fu et al. 2015).

Recently, it was shown that purified *M. tuberculosis* FtsE binds ATP and that FtsE dimers have a relatively low ATPase activity (Mir et al. 2006, 2015). Purified *M*. *tuberculosis* FtsE required denaturation and refolding during purification and formed an intersubunit disulfide bond that stabilized the FtsE dimer and prevented precipitation out of solution (Mir et al. 2015). To date, it has not been possible to purify full‐length *E. coli* or *M. tuberculosis* FtsX, because overexpression of these two proteins is toxic to *E. coli* (de Leeuw et al. 1999; Mir et al. 2006, 2015). In this paper, we report the first purification and biochemical characterization of *S. pneumoniae* FtsE and full‐length FtsX, including initial attempts to isolate and reconstitute the FtsEX:PcsB complex biochemically.

## Materials and Methods

### Plasmids and strains

Bacterial strains and plasmids used in this study are listed in Table S1. *Escherichia coli* strains BL21AI (Narayanan et al. 2011) and BL21DE3 (Novagen, CA) were used for construction of strains and expression of proteins. Depending on the plasmid, kanamycin (25–50 *μ*g/mL), ampicillin (100 *μ*g/mL), and/or chloramphenicol (20–34 *μ*g/mL) were used to select for transformants in *E. coli*. Vectors used in this study are described in the following sources: pETCT‐GFP‐His_8_ (Narayanan et al. 2011); pET22b‐His_6_ (Novagen/EMD Millipore, MA, USA); pEV‐L4‐His_6_‐GFP (provided by Professor Jue Chen's laboratory); and pACYC‐Duet (Novagen/EMD Millipore, MA, USA). Inserts into plasmids were made by standard cloning techniques using the restriction sites listed in Table S1. Plasmids pET22b‐PcsB^ΔN27^, pET22b‐PcsB‐CC^28−259^, and pET22b‐PcsB^271−392^ were described in (Sham et al. 2011). His_8_ and His_6_ tags are abbreviated as “His” throughout the text.

### Expression of the FtsX‐GFP‐His fusion construct

IU6892 (BL21AI pETCT‐*ftsX*‐GFP‐His) was grown in 50 mL LB broth to OD_600_ = 0.5, and temperature and inducer concentrations described in the text were used to express FtsX‐GFP‐His. After induction overnight, cells were lysed and FtsX‐GFP‐His expression was monitored by GFP fluorescence using a fluorescence plate reader (Spectramax 5 plate reader, Molecular Devices, CA; Ex = 395 nm; Em = 509 nm). Induced cells were collected by centrifugation at 4000*g* at 4°C for 10 min. Pellets from 1 L cultures were suspended in 35 mL lysis buffer (50 mmol/L Tris‐HCl pH 8.0, 200 mmol/L NaCl, 15% glycerol) and lysed by sonication (Branson digital sonifier; 1/8″ tapered microtip; pulse on = 0.5 sec/pulse off = 1.5 sec for 2 min; amplitude = 20%). Cell lysates were centrifuged in 1.5 mL polypropylene tubes at 100,000*g* at 4°C for 1 h to pellet membranes. Membranes were resuspended in lysis buffer additionally containing 1% (wt/vol) n‐dodecyl‐*β*‐d‐maltoside (DDM) (Anatrace, Inc., OH. USA) for 2 h to solubilize membranes and centrifuged at 100,000*g* at 4°C for 30 min to pellet the insoluble material. The insoluble fraction was discarded and solubilized membranes were run on SDS‐PAGE. The position of FtsX‐GFP‐His on the SDS‐PAGE gel was determined by western blotting using anti‐GFP antibody. Different detergents were tested for optimal extraction of FtsX‐GFP‐His as described in the Results and Discussion.

### Optimization of purification of FtsX‐GFP‐His

IU6892 (BL21AI pETCT‐*ftsX*‐GFP‐His) was grown in Terrific Broth (Research Products International, IL, USA) at 37°C with shaking at 250 rpm to OD_600_ = 0.5. Cells were induced with 1 mmol/L IPTG at 25°C and grown overnight. The next day, cultures were harvested by centrifugation at 4000*g* at 4°C for 10 min. Cell pellets were resuspended in Buffer X (50 mmol/L Tris‐HCl pH 8.0, 200 mmol/L NaCl) containing 15% glycerol, 1 mmol/L PMSF, and DNase I (Roche Diagnostics Corp., IN, USA) to 5 *μ*g/mL. Cell suspensions were run through a French Press at 18,000 psi once. Lysates were centrifuged at 100,000*g* at 4°C for 1 h to pellet cell membranes. Membranes were resuspended at a protein concentration of 3–5 mg/mL, determined by the Bradford protein assay (BioRad, CA, USA), and DDM was added slowly to the membrane suspension to a concentration of 0.5% (wt/vol). Solubilized membranes were nutated for 2 h, and insoluble material was removed by centrifugation at 100,000*g* at 4°C for 30 min.

Membrane supernates were passed through a Co^2+^ resin gravity column (2 mL resin slurry per L of processed cells) (Takara/Clontech, CA, USA) equilibrated with Buffer X containing various detergent mixtures described in Results and Discussion at concentrations ≥1X their critical micellar concentration (CMC). Glycerol concentrations were also varied to optimize recovery. After loading, the column was washed with Buffer X + detergents + glycerol containing 10 mmol/L imidazole until protein concentration in the flow through was too low to be detected by Bradford protein assays (BioRad, CA, USA). FtsX‐GFP‐His was then eluted with the Buffer X + detergents + glycerol containing 200 mmol/L imidazole. Fractions containing FtsX‐GFP‐His were pooled, concentrated using a Millipore ultraconcentrator (cutoff = 100 kDa, (EMD Millipore, MA, USA)), and loaded onto a Superdex 200 30/100 column (GE Healthcare Life Sciences, PA, USA) connected to an HPLC with an in‐line fluorescence detector (Shimadzu Corp., MD, USA). The sizing column was equilibrated with Buffer X + detergents + glycerol, and elution of FtsX‐GFP‐His was monitored by fluorescence detection (Ex = 395 nm; Em = 510 nm). These elution conditions producing qualitatively Gaussian peaks were considered for incorporation into a final purification scheme as described in (Kawate and Gouaux 2006). All results were reproduced at least twice, and representative chromatograms are shown.

### Western blotting

Western blotting was done by standard methods (see (Sham et al. 2011)). Proteins on SDS‐PAGE gels were transferred to nitrocellulose membranes in wet conditions (20 mmol/L Tris base, 150 mmol/L glycine) using an electric field of 12 mV for 1 h. Blots were blocked with 5% (wt/vol) nonfat milk in TNT transfer buffer (20 mmol/L Tris‐HCl, pH 7.5, 500 mmol/L NaCl, 0.05% (vol/vol) Tween 20) overnight at room temperature. For GFP‐tagged or His‐tagged proteins, the primary antibody was mouse anti‐GFP or anti‐His antibody, respectively (Abcam, Cambridge, UK). The secondary antibody was anti‐mouse AlexaFluor‐683 antibody (Molecular Probes/ThermoFisher, IL, USA). Blots were imaged by using an Odyssey Infra‐red Imaging System (LI‐COR Biosciences, NE, USA).

### Purification of FtsX‐His

The optimum conditions for FtsX‐GFP‐His purification were used for FtsX‐His purification from strain IU6942 (BL21DE3/pET22b‐*ftsX‐*His). Membrane purification and solubilization were performed as described above for optimization of FtsX‐GFP‐His purification. Membrane supernates were loaded onto a gravity column containing Co^2+^‐affinity resin (2 mL resin slurry per L of processed cells) (Takara/Clontech, CA, USA), equilibrated with Buffer X‐opt (Buffer X containing 5% glycerol, 0.02% (wt/vol) DDM, and 0.02% (wt/vol) C_12_E_8_ detergent). Washes were performed with Buffer X‐opt (15–20 column volumes) containing 10 mmol/L imidazole until the protein concentration of flow‐through samples was undetectable by the Bradford protein assay. FtsX‐His was eluted by Buffer X‐opt containing 200 mmol/L imidazole and FtsX‐His‐containing fractions were pooled and concentrated as described above. FtsX‐His was further purified by size‐exclusion chromatography on a Hi‐Load Superdex 200 16/60 column (GE Healthcare) equilibrated and eluted with Buffer X‐opt. Fractions containing FtsX‐His were identified by SDS‐PAGE, pooled, and stored at −80°C at a protein concentration = 10–30 *μ*mol/L. FtsX‐His concentration was determined by A_280_ using an extinction coefficient of *ε *= 29,910 M^−1 ^cm^−1^ calculated by the ExPASy ProtParam program (Gasteiger et al. 2003).

### Incorporation of FtsX‐His into nanodiscs

Membrane scaffolding protein MSPE3D1‐His was purified and cleaved by TEV protease to remove the His tag as described previously (Alvarez et al. 2010). Purified FtsX‐His was reconstituted into nanodiscs using the procedure described in (Alvarez et al. 2010). Briefly, powdered soybean lipids (P5638, Sigma‐Aldrich, MO, USA) were dissolved in Buffer N (20 mmol/L Tris‐HCl pH 8.0, 100 mmol/L NaCl) in a glass tube to prepare a stock solution of 50 mg/mL, which was clarified by sonication until translucent. To optimize nanodisc formation, sodium cholate (Anatrace, Inc., OH, USA), soybean lipids, MSPE3D1, and purified 10–12 *μ*mol/L FtsX‐His in Buffer X‐opt were mixed together in different ratios in a volume of 450 *μ*L and incubated at room temperature for 1 h with gentle rocking. Bio‐beads SM‐2 (0.3 mg, BioRad, CA, USA) were hydrated by washing in methanol, water, and Buffer N, each three times. Hydrated Bio‐beads were added to the nanodisc mixture to initiate nanodisc assembly. The resulting mixture was incubated at room temperature for 3 h with gentle rocking. Bio‐beads were removed, and the sample was loaded on 300 *μ*L of Ni‐NTA resin (Qiagen, CA, USA) contained in a 0.5 mL column equilibrated with buffer N. The column was washed four times with 1 mL of Buffer N containing 10 mmol/L imidazole to remove empty nanodiscs. FtsX‐His nanodiscs were eluted by washing with Buffer N containing 200 mmol/L imidazole. Fractions containing FtsX‐His nanodiscs were pooled and purified further by size‐exclusion chromatography (Superdex 200 30/100) as described before (Alvarez et al. 2010). Reconstitution of FtsX‐His into nanodiscs was confirmed by detecting both MSPE3D1 and FtsX‐His bands in SDS‐PAGE and by electron microscopy and light scattering (see Results and Discussion). Ratios of FtsX‐His to MSPE3D1 = 1:10 and MSPE3D1 to soybean lipid = 1:80 optimally allowed incorporation of FtsX‐His into nanodiscs (see Results and Discussion).

### Protein crosslinking to detect FtsX‐His dimers

Crosslinking with glutaraldehyde was done as described in (Foster et al. 2004). Purified FtsX‐His in Buffer X‐opt was exchanged into a buffer lacking Tris‐HCl (50 mmol/L HEPES pH 8.0, 200 mmol/L NaCl, 5% (vol/vol) glycerol, 0.02% (wt/vol) DDM, 0.02% (wt/vol) C_12_E_8_) by running on a Superdex 200 30/100 column. Purified FtsX‐His (5–10 *μ*mol/L) was treated with glutaraldehyde (0.5–2.0 mmol/L) in a 20 *μ*L volume for 30–45 min at room temperature. Reaction was stopped by adding Tris‐containing 5X SDS sample buffer, and samples were run on a 12% SDS‐PAGE gel. Crosslinked bands were visualized by Coomassie blue staining.

### Expression and purification of FtsE‐His

Strain IU4340 (BL21DE3/pET22b‐*ftsE*‐His) was grown and FtsE‐His expression induced as described above for the purification of FtsX‐His. Cell pellets were resuspended in Buffer E (20 mmol/L Tris‐HCl pH 8.0, 100 mmol/L NaCl) containing 1 mmol/L PMSF and DNase I (Roche Diagnostics Corp., IN, USA) to 5 *μg*/mL. Cell suspensions were run through a French Press at 18,000 psi once. Cell lysates were centrifuged at 10,000*g* at 4°C for 30 min. Supernates were loaded by gravity onto a Ni‐NTA column (Qiagen, CA, USA) equilibrated with Buffer E. The column was washed with 10 mmol/L imidazole in Buffer E until protein could not be detected by the Bradford protein assay, and FtsE‐His was eluted with 200 mmol/L imidazole in Buffer E. Eluted FtsE‐His was concentrated using a Millipore ultra‐concentrator (cutoff = 10 kDa) and loaded onto a Hi‐Load Superdex 200 16/60 column (GE Healthcare Life Sciences, PA, USA), equilibrated with Buffer E. Following elution in Buffer E, fractions containing FtsE‐His were located by SDS‐PAGE and pooled. FtsE‐His concentration determined by A_280_ using an extinction coefficient of *ε *= 17,420 M^−1 ^cm^−1^ calculated by the ExPASy ProtParam program (Gasteiger et al. 2003).

### TNP‐ATP binding and ATPase assays of FtsE‐His

Fluorescence measurements were carried on Fluoromax‐3 spectrofluorimeter (Horiba Scientific). TNP‐ATP binding assays were conducted as described previously (Stewart et al. 1998), with excitation at 410 nm and emission scans recorded from 450 to 600 nm (slit width = 3 nm; integration time interval = 0.1 sec). FtsE‐His at a constant concentration (4.5 *μ*mol/L) in 20 mmol/L Tris‐HCl pH 8.0, 100 mmol/L NaCl was titrated with increasing concentrations of TNP‐ATP from 0 to 400 *μ*mol/L in a volume of 2.5 mL. Fluorescence intensity at 550 nm was plotted against increasing concentrations of TNP‐ATP to determine the affinity constant (K_d_) between FtsE‐His and TNP‐ATP. The concentration of TNP‐ATP at half saturation of fluorescence intensity was reported as the K_d_.

ATP hydrolase activity of purified FtsE‐His was determined by a pyruvate kinase and lactate dehydrogenase coupled enzyme assay as described previously (Orelle et al. 2008). Reaction mixtures contained 50 mmol/L HEPES/KOH pH 8.0, 10 mmol/L MgCl_2_, 4 mmol/L phosphoenolpyruvate, 60 *μ*g pyruvate kinase per mL, 32 *μ*g lactate dehydrogenase per mL, 0.3 mmol/L NADH, and 1.5 mmol/L ATP. A typical reaction was carried out in 100 *μ*L with 20 *μ*g of FtsE‐His and absorbance was recorded using 8453 UV‐Vis Diode Array System (Agilent Technologies, CA, USA) at 25°C for 10 min. Experiments were performed three times from the same protein preparations. Additional proteins were added to ATPase assays at the molar ratios indicated in Table 2. The reconstituted maltose transporter was used as a positive control for ATPase activity (Orelle et al. 2008).

### Expression and purification of PcsB^ΔN27^‐His (intact PcsB‐His lacking its signal sequence), PcsB‐CC^28−259^‐His (CC^PcsB^‐His domain), and PcsB‐CHAP^279−392^‐His (CHAP^PcsB^‐His domain)

Strains IU1617 (pET22b‐*pcsB* ^ΔN27^‐His), IU4561 (pET22b‐*pcsB* ^28−259^ (CC)‐His), and IU1791 (pET22b‐*pcsB* ^271−392^ (CHAP)‐His) were grown at 37°C with shaking at 250 rpm to OD_600_ = 0.6. Proteins were induced with 1 mmol/L IPTG, and growth of cultures was continued for 4 h. Cells were harvested and lysed as described above for FtsE‐His using lysis/purification Buffer E (20 mmol/L Tris‐HCl pH 8.0, 100 mmol/L NaCl), except that 5 mmol/L *β*‐mercaptoethanol was added to Buffer E during purification of the PcsB‐His and CHAP^PcsB^‐His to keep cysteines reduced. Purification was carried out as described above for FtsE‐His using a Ni‐NTA gravity column and a Hi‐Load 16/60 Superdex 200 column. Eluted proteins were located by SDS‐PAGE, and concentrations were determined by using the following extinction coefficients calculated by the ExPASy ProtParam program (Gasteiger et al. 2003): PcsB‐His (*ε *= 45,660 M^−1 ^cm^−1^); CC^PcsB^‐His (2980 M^−1 ^cm^−1^); and CHAP^PcsB^‐His (43,555 M^−1 ^cm^−1^).

### Association studies between FtsX‐His, FtsE‐His, and PcsB‐His

Combinations of purified FtsX‐His + FtsE‐His (1:1 molar ratios, where the amount of FtsE‐His was 2.1 nmoles) and FtsX‐His + FtsE‐His + PcsB‐His (1:1:2 molar ratios) were mixed together in 50 mmol/L Tris‐HCl pH 8.0, 200 mmol/L NaCl, 5% (vol/vol) glycerol, 0.02% (wt/vol) DDM, and 0.02% (wt/vol) C_12_E_8_ in the absence or presence of vanadate trapping reagents (0.5 mmol/L vanadate, 5.0 mmol/L MgCl_2_ and 5.0 mmol/L ATP) (Sharma and Davidson 2000; Chen et al. 2001) in a final volume of ≈120 *μ*L. Mixtures were incubated overnight at 4°C. The next day, samples were run on a Superdex 200 30/100 size‐exclusion column in the same buffer and peaks were detected by in‐line A_280_ detection. Fractions were collected and run on SDS‐PAGE to test for complex formation.

Pull down experiments of FtsE‐His + FtsX or FtsE + FtsX‐His coexpressed in *E. coli* strains IU10588 (BL21DE3/pET22b *ftsE*‐His_6_ + pACYC‐Duet‐*ftsX*) and IU10589 (BL21DE3/pET22b‐*ftsX*‐His_6_ + pACYC‐Duet‐*ftsE*), respectively (Table S1), were done by following the purification scheme for FtsX‐His described above through the Co^2+^ affinity chromatography step. Cofactors (5 mmol/L ATP; 5 mmol/L ATP + 5 mmol/L Mg^2+^; 5 mmol/L ADP + 5 mmol/L Mg^2+^; 5 mmol/L ATP + 5 mmol/L Mg^2+^ + 0.5 mmol/L VO_4_^2−^) were added to cell membranes before solubilization with DDM, and cofactors were kept at the same concentrations in subsequent steps of the purification.

### Liposome sedimentation binding assays

Liposomes were formed by mixing phospholipids (PC = 1‐palmitoyl‐2‐oleoylphosphatidylcholine; PG = 1‐palmitoyl‐2‐oleoyl‐sn‐glycero‐3‐phosphoglycerol; PE = 1‐palmitoyl‐2‐oleoyl‐sn‐glycerol‐3‐phosphoethanolamine; *E.coli* polar lipids; *E.coli* total lipids; Avanti polar lipids) in different ratios in chloroform, drying the mixtures with argon gas, and incubating the dried samples in vacuum overnight at room temperature. Dried lipids were suspended in 20 mmol/L Tris‐HCl pH 8.0 to a concentration of 25 mg/mL, and the mixtures were placed in a sonicator bath until the solution became translucent. In sedimentation binding assays, the total amount of lipids was 5.0 mg in 100 *μ*L of mixture, unless indicated otherwise. PcsB‐His, CC^PcsB^‐His, or CHAP^PcsB^‐His at constant concentrations of 50–100 *μ*M was added to liposome mixtures, and samples were incubated for 1 h at room temperature. Mixtures were centrifuged at 100,000*g* for 1 h at 4°C, and pellets were washed once with 20 mmol/L Tris‐HCl pH 8.0. Supernates and resuspended pellets were run on SDS‐PAGE, and gels were stained with Coomassie dye. For binding assays, a constant concentration of PcsB‐His or CC^PcsB^‐His was titrated with liposomes containing increasing total amounts (0.5–5.0 mg) of a 50% PE + 50% PC mixture, and band intensities on gels were quantitated by gel densitometry.

### Computer generated structure of PcsB

The structure of PcsB was previously solved (Bartual et al. 2014). The structure was downloaded from the RCSB Protein Data bank website (PDB ID: 4CGK). PcsB was visualized via PyMOL (Schrödinger, LLC) to observe the structure. PyMOL was modified using the Color h script adapted from PyMOLWiki and reference (Eisenberg et al. 1984). Hydrophobicity of the structure was observed using the Color h script, with the surface mode on, and glutamate residues were colored yellow.

## Results and Discussion

### Expression and purification of full‐length pneumococcal FtsX‐His

As a first step in characterizing the pneumococcal FtsEX:PcsB complex, we worked out and optimized conditions for FtsX purification. Previous work demonstrated that overexpression of FtsX from *Escherichia coli* or *Mycobacterium tuberculosis* is toxic to *E. coli* cells, thereby hindering FtsX purification (de Leeuw et al. 1999; Mir et al. 2006, 2015). To overcome this potential problem, we first expressed FtsX fusions in *E. coli* strain BL21AI, which was used previously to express toxic membrane proteins at high yields from arabinose‐inducible plasmid constructs (Narayanan et al. 2011). We fused FtsX at its C‐terminus to a GFP‐His tag in vector pETCT‐GFP‐His (Table S1), which allows tunable expression of fusion proteins from a T7 promoter that is stimulated by arabinose addition, but repressed in the absence of IPTG (Narayanan et al. 2011). To test for expression of FtsX fusion proteins, membranes were pelleted from lysed cells and solubilized with detergent as described in Materials and Methods. Several detergents were initially screened for optimized extraction of FtsX‐GFP‐His fusion protein from membranes into a solubilized fraction, and 0.5–2% (wt/vol) n‐Dodecyl‐*β*‐D‐maltoside (DDM) was found to be the best in terms of cost and availability (Table S2). The amount of solubilized FtsX‐GFP‐His extracted from membranes of cells subjected to different inducing conditions was determined by fluorescence intensity and western blotting with anti‐GFP antibody (Table 1; Fig. S1).

**Table 1:** Maximal expression in membranes and detergent solubilized FtsX‐GFP‐His under full induction conditions implies that FtsX‐GFP‐His is not toxic to *E. coli* a

Inducer^b^	Fluorescence intensity in membrane resuspension (AU)^c^	Fluorescence intensity in DDM‐extracted membrane supernate (AU)^d^
None	293	127
0.001% arabinose	586	295
0.01% arabinose	446	339
0.02% arabinose	547	460
0.05% arabinose	465	408
0.1% arabinose	546	443
0.5 mmol/L IPTG	338	305
0.5 mmol/L IPTG + 0.01% arabinose	4160	2850

a Strain IU6892 (BL21AI (pETCT‐*ftsX*‐GFP‐His)) was grown and induced as described in Materials and Methods.

b Addition of 0.5 mmol/L IPTG + 0.01% arabinose causes maximal transcriptional induction from this plasmid (Narayanan et al. 2011).

c AU, arbitrary unit.

d FtsX‐GFP‐His was extracted from membranes with 1% (wt/vol) DDM as described in Materials and Methods.

Unexpectedly, we observed maximum recovery of FtsX‐GFP‐His when the promoter was fully induced by the addition of 0.01% (wt/vol) arabinose + 0.5 mmol/L IPTG (Table 1, last column; Fig. S1, lane 8), indicating that pneumococcal FtsX‐GFP‐His expression is not toxic to *E. coli* cells. We found that N‐terminal‐tagged His‐GFP‐FtsX could also be overexpressed in strain BL21AI from vector pEV‐L4 (Table S1), but its recovery from membranes was ≈10‐fold lower than that of C‐terminal FtsX‐GFP‐His (data not shown); hence, we focused the remainder of this study on the C‐terminal fusion FtsX‐GFP‐His. Because pneumococcal FtsX overexpression was not toxic to *E. coli* cells (Table 1), we simplified the overexpression system by moving plasmid pETCT‐*ftsX*‐GFP‐His into *E. coli* strain BL21DE3 (Table S1) and inducing FtsX‐GFP‐His from the T7 promoter by IPTG addition. Optimal recovery of FtsX‐GFP‐His was obtained with 1.0 mmol/L IPTG at 25°C (Table S3). Later experiments confirmed a lack of pneumococcal FtsX‐His toxicity by the slow growth of cultures after induction with IPTG (see below; Fig. S2).

**Figure 2:**
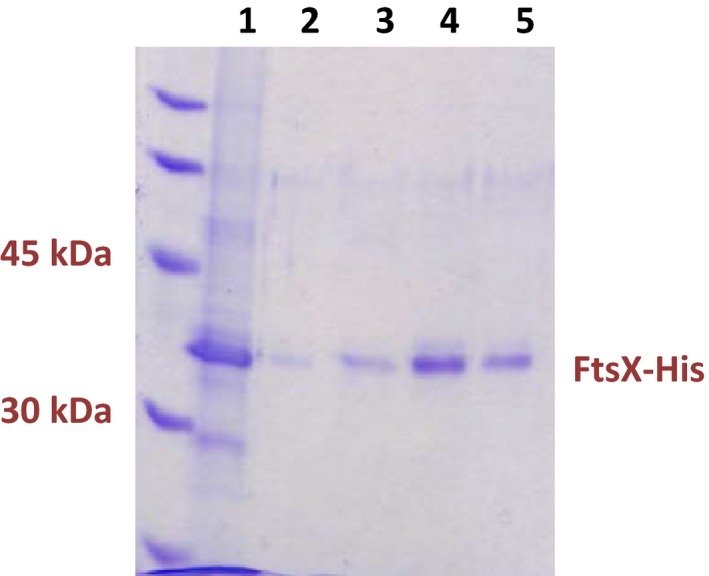
Purification of FtsX‐His. SDS‐PAGE: lane 1, cobalt column affinity‐purified FtsX‐His; Lanes 2–5, fractions from gel filtration after affinity‐purified FtsX‐His was loaded on a Hi Load Superdex 200 16/60 column that was equilibrated and eluted with 50 mmol/L Tris‐HCl, pH 8.0, 200 mmol/L NaCl, 5% (vol/vol) glycerol, 0.02% (wt/vol) DDM, and 0.02% (wt/vol) C_12_E_8_. The FtsX‐His band at 34 kDa and the standards ladder are indicated in the SDS‐PAGE gel stained with Coomassie blue. Overexpression, affinity purification, gel filtration, and storage of FtsX‐His are described in Materials and Methods.

We optimized purification of FtsX‐GFP‐His and then FtsX‐His. Fluorescence‐detection size‐exclusion chromatography (FSEC) was used to screen variables, including type and amount of detergent and glycerol, with the goal of obtaining a homogenous preparation of FtsX‐GFP‐His (Fig. S3) (Kawate and Gouaux 2006). After extraction of FtsX‐GFP‐His from membranes with buffer containing 0.5% (wt/vol) DDM (see Materials and Methods), FtsX‐GFP‐His was optimally stabilized in micelles by a mixture of two detergents (0.2% (wt/vol) DDM and 0.02% (wt/vol) C_12_E_8_) in buffer containing 5% (vol/vol) glycerol (Fig. S3), which was used in subsequent purifications. We optimized purification of FtsX‐GFP‐His by cobalt‐resin affinity chromatography followed by size‐exclusion chromatography as detailed in Materials and Methods. The optimized conditions for FtsX‐GFP‐His purification were then used to purify FtsX‐His to near homogeneity as a single 34 kDa band, matching its predicted molecular mass, on SDS‐PAGE (Fig. 2). Large‐scale purification yielded 0.5 mg of purified FtsX‐His protein from 1 L of induced culture in Terrific Broth. We conclude that pneumococcal FtsX‐His can be overexpressed and purified to near homogeneity, unlike FtsX homologs from some other bacterial species.

### FtsX‐His forms dimers in reconstituted membrane nanodiscs and in detergent micelles

**Figure 3:**
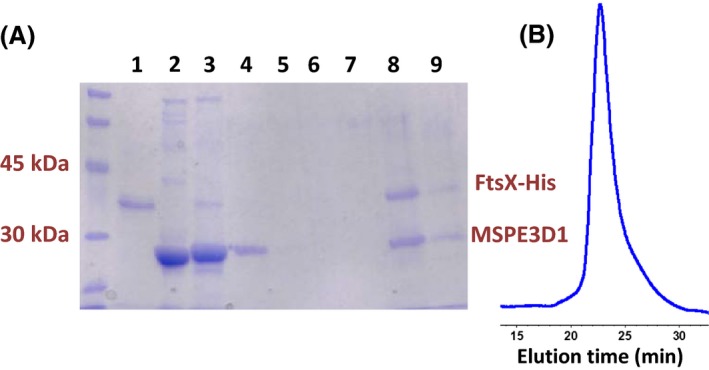
Reconstitution of purified FtsX‐His in nanodiscs. A. SDS‐PAGE of fractions from purification of reconstituted FtsX‐His nanodiscs. Lane 1, FtsX‐His alone; lane 2, MSPE3D1 alone; lane 3, flow through of nanodisc reconstitution from Ni‐NTA column; lane 4, wash 1; lane 5, wash 2; lane 6, wash 3; lane 7, wash 4; lane 8, Ni‐NTA‐column affinity‐purified nanodiscs; lane 9, nanodiscs purified by affinity chromatography followed by gel filtration. Purification of FtsX‐His and nanodisc reconstitution are described in Materials and Methods. B. Elution profile of FtsX‐His nanodiscs in analytical size‐exclusion chromatography (Superdex 200 30/100; buffer = 20 mmol/L Tris‐HCl; 8.0, 100 mmol/L NaCl; flow rate = 0.5 mL per min; A_280_ detection). Nanodiscs eluted at 23.5 min, corresponding to a volume of ≈11.8 mL. Minimal additional peaks were detected elsewhere in chromatograms (data not shown). The experiment was performed independently three times with similar results.

We next incorporated purified FtsX‐His into nanodiscs, which are artificial membrane models that consist of a patch of lipid bilayer surrounded by a membrane scaffolding protein (Nath et al. 2007; Borch and Hamann 2009). We expect that FtsX incorporated into nanodiscs would expose the hydrophilic extracellular ECL1 and ECL2 domains and the cytoplasmic FtsE‐binding domains (Fig. 1) to the aqueous medium, thereby, allowing interaction studies (Nath et al. 2007). We used the method established previously in the Davidson laboratory for nanodisc reconstitution with the MSPE3D1 membrane scaffolding protein, which forms nanodiscs with diameters ≈ 13 nm (Nath et al. 2007) (see Materials and Methods). MSPE3D1‐His was purified and treated with TEV protease to remove its His tag as described before (Alvarez et al. 2010). Reconstitution of FtsX‐His nanodiscs particles was optimized by varying the ratios of FtsX‐His to MSPE3D1 (1:2, 1:5, 1:10, 1:20) and MSPE3D1 to lipid (1:50, 1:80). Ratios of FtsX‐His to MSPE3D1 = 1:10 and MSPE3D1 to lipid = 1:80 optimally allowed incorporation of FtsX‐His into nanodiscs. Reconstituted FtsX‐His nanodiscs were purified by Ni‐NTA affinity chromatography and the eluted fraction was further purified by size‐exclusion chromatography (Fig. 3).

The presence of both MSPE3D1 and FtsX‐His in the eluted and size‐exclusion purified fractions confirmed the reconstitution of FtsX‐His in nanodiscs (Fig. 3A). The purified FtsX‐His nanodisc eluted as a single symmetrical peak from the analytical size‐exclusion column, confirming the homogeneity of the nanodisc preparation (Fig. 3B). Moreover, the nanodiscs containing FtsX‐His eluted from the size‐exclusion column at an elution volume (≈11.8 mL) comparable to other protein‐loaded nanodiscs containing MSPE3D1 reported in the literature (Denisov et al. 2004; Bayburt et al. 2006; Borch and Hamann 2009). Transmission electron microscopy of negatively stained samples and dynamic light scattering measurements confirmed that the purified FtsX‐His nanodiscs were largely homogenous with a diameter of ≈ 18 nm (data not shown).

FtsX shares structural features with the transmembrane proteins of ABC transporters (Fig. 1) (Schmidt et al. 2004; Weiss 2004; Arends et al. 2009). Consequently, we anticipated that pneumococcal FtsX would form dimers. The oligomeric state of some membrane proteins has been determined in reconstituted nanodiscs (e.g., see (Bayburt et al. 2006)). Each nanodisc particle contains at least two molecules of membrane scaffold protein (Nath et al. 2007). We used densitometry to quantitate the relative intensities of the Coomassie‐stained MSPE3D1 (26.0 kDa) and FtsX‐His (34.0 kDa) bands in eluted and size‐exclusion purified nanodiscs (Fig. 3A) (see Materials and Methods) (Alvarez et al. 2010). FtsX‐His and MSPE3D1 were present in approximately equal amounts on staining. Because reconstituted FtsX‐His nanodiscs were purified by Ni‐NTA affinity chromatography (see above), the fraction of empty nanodiscs should be negligible. Assuming two MSPE3D1 molecules per nanodisc, this result is consistent with loading of ≈1.5 FtsX‐His molecules per nanodisc on average under these conditions, suggesting a mixed population of nanodiscs containing monomer or dimer FtsX‐His.

**Figure 4:**
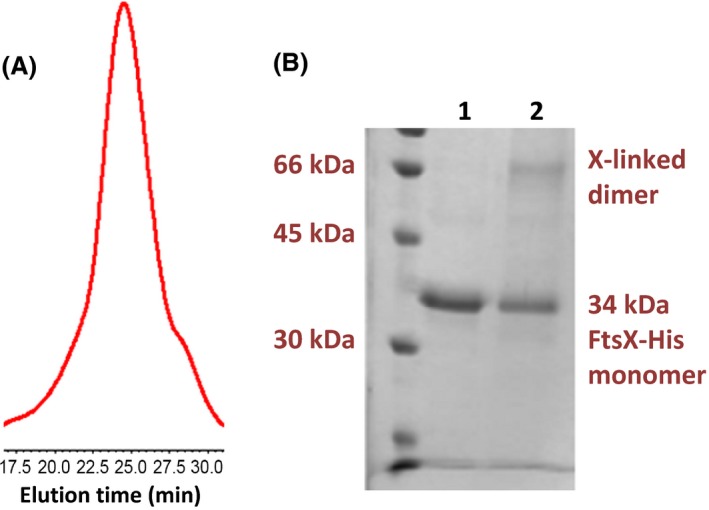
FtsX‐His forms a dimer in detergent micelles. (A) Elution profile of FtsX‐His in analytical size exclusion chromatography (Superdex 200 30/100; Buffer X‐opt = 50 mmol/L Tris‐HCl pH 8.0, 200 mmol/L NaCl, 5% (vol/vol) glycerol, 0.02% (wt/vol) DDM, 0.02% (wt/vol) C_12_E_8_; flow rate = 0.5 mL per min). Numbers on X axis in chromatogram represent elution time in minutes. Minimal additional peaks were detected elsewhere in chromatograms (data not shown). (B) Crosslinking profile of FtsX‐His with glutaraldehyde in 50 mmol/L HEPES pH 8.0, 200 mmol/L NaCl, 5% (vol/vol) glycerol, 0.02% (wt/vol) DDM, 0.02% (wt/vol) C_12_E_8_. SDS‐PAGE: lane 1, FtsX‐His, no glutaraldehyde; lane 2, FtsX‐His crosslinked with 2.0 mmol/L glutaraldehyde as described in Materials and Methods. The FtsX‐His monomer band at 34 kDa and the crosslinked dimer band near 66 kDa is indicated. The experiment was performed several times with similar results. At this level of crosslinking, additional high‐molecular weight bands were not detected near the tops of gels (data not shown).

FtsX‐His also formed dimeric species in detergent micelles. FtsX‐His dissolved in a mixture of nonionic detergents and glycerol eluted as a single symmetrical peak in analytical size‐exclusion chromatography (Fig. 4A). The elution volume of 12 mL corresponds to a molecular mass >200 kDa, based on calibration of the column, which is considerably larger than the 34 kDa molecular mass of the FtsX‐His monomer (Fig. 2). However, FtsX‐His elution will depend on FtsX‐His oligomerization and detergent binding, which are unknown. To probe for FtsX‐His dimerization in the micelle mixture, we used crosslinking with glutaraldehyde followed by SDS‐PAGE (Foster et al. 2004) (see Materials and Methods). A band with molecular mass of ≈66 kDa, consistent with an FtsX‐His dimer, was detected in the crosslinked sample and was absent in the non‐crosslinked sample (Fig. 4B). No other X‐linked species was visible on this gel, and comparable reaction conditions failed to crosslink purified FtsE‐His into a dimer (see below; data not shown). Taken together, we conclude that FtsX‐His forms dimers in detergent micelles and in reconstituted nanodiscs, consistent with other transmembrane domain proteins from ABC transporters (see [Davidson et al. 2008; Rees et al. 2009]).

### Purified FtsE‐His binds ATP, and FtsE‐His preparations exhibit low ATPase activity

**Figure 5:**
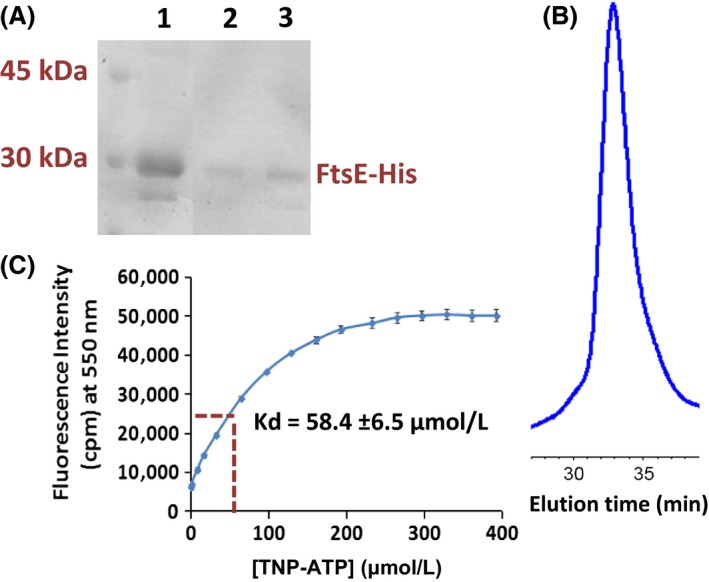
Purification of FtsE‐His and determination of ATP binding. (A) SDS‐PAGE: lane 1, Ni‐NTA‐column affinity‐purified FtsE‐His, which was further purified by size‐exclusion chromatogrphy; lanes 2 and 3, fractions from size‐exclusion chromatography of affinity‐purified FtsE‐His. Additional bands were not visible in these lanes elsewhere in the gels (data not shown) A section of the gel lacking samples was removed in the image between lanes 1 and 2. (B). Size‐exclusion chromatogram (Superdex 200 30/100 in 20 mmol/L Tris pH 8.0, 100 mmol/L NaCl; flow rate = 0.5 mL per min; A_280_ detection) of purified FtsE‐His. The elution time of 33 min corresponds to an elution volume of 16.5 mL. Minimal additional peaks were detected elsewhere in chromatograms (data not shown). FtsE‐His overexpression and purification were carried out as described in Materials and Methods. (C) TNP‐ATP fluorescence binding assay of purified FtsE‐His. The assay and titration procedure are described in Materials and Methods. The K_d_ determined by the concentration of TNP‐ATP at half saturation of fluorescence intensity is indicated. Data points were averaged from two measurements.

Pneumococcal FtsE has been hypothesized to act as an ATPase in a complex with FtsX (Fig. 1). Amino acid changes in the Walker box motifs of pneumococcal FtsE are not tolerated and led to growth stoppage and cell death (Sham et al. 2013). The FtsE homolog of *M. tuberculosis* has been purified and shown to bind ATP weakly and to have low ATPase activity. Purification of FtsE_Mtb_ presented numerous challenges, because the protein had to be renatured and dimerized by formation of an intersubunit cystine disulfide bond (Mir et al. 2006, 2015). The single Cys residue present in FtsE_Mtb_, which is not required for dimerization nor catalysis (Mir et al. 2015), is absent in FtsE_Spn_. We found that pneumococcal FtsE‐His could be readily overexpressed in *E. coli* and purified without a renaturation step (see Materials and Methods). FtsE‐His was purified by Ni‐NTA affinity chromatography and size‐exclusion chromatography (Fig. 5A and 5B). In size‐exclusion chromatography, purified FtsE‐His eluted as a single symmetrical peak with a molecular mass of ≈45 kDa, which is larger than the predicted monomer molecular mass of ≈26 kDa (Fig. 5B). However, an FtsE‐His dimer was not detected by glutaraldehyde crosslinking (see above), and definitive support for FtsE‐His dimerization was not obtained in the conditions tested. In Coomassie‐stained SDS‐PAGE gels, two bands were detected in FtsE‐His preparations; a major band at ≈30 kDa and a minor band at ≈26 kDa (Fig. 5A and S4). Western blots with anti‐His‐tag antibody confirmed that the ≈30 kDa band corresponds to FtsE‐His (Fig. S4). The yield of purified FtsE‐His was ≈2 mg/L of culture induced in Terrific Broth.

We assessed ATP binding to purified FtsE‐His by binding of the fluorescent ATP analog, TNP‐ATP (see Materials and Methods) (Stewart et al. 1998). TNP‐ATP fluorescence increases and shifts to 550 nm when it binds to proteins in neutral or basic buffers. Titration assays were carried out with a constant concentration of FtsE‐His (4.5 *μ*mol/L) and increasing concentrations of TNP‐ATP (Fig. 5C). Fluorescence of TNP‐ATP at 550 nm increased hyperbolically with increasing concentrations of TNP‐ATP. The K_d_ for TNP‐ATP binding of FtsE‐His was calculated to be 58.4 ± 6.5 *μ*mol/L (Fig. 5C), which is comparable to the K_d_(ATP) of other ATPase subunits of ABC transporters (see [Davidson et al. 2008]). Circular dichroism spectroscopy showed that FtsE‐His contains ≈20% *α*‐helical structure, which was not significantly changed by increasing ATP concentrations to 300 *μ*mol/L (data not shown). Last, we used an enzyme‐coupled assay to determine the ATPase activity of purified FtsE‐His alone or with purified FtsX‐His and PcsB‐His in detergent micelles or FtsX‐His reconstituted into nanodiscs (Table 2). In all conditions, a low ATPase activity was detected for FtsE‐His preparations above background; however, FtsE‐His ATPase activity was <100‐fold lower compared to that of the reconstituted maltose ABC transporter (Orelle et al. 2008; Alvarez et al. 2010). FtsE‐His ATPase was not significantly stimulated by addition of FtsX‐His or FtsX‐His and PcsB‐His in micelles or nanodiscs (Table 2). We conclude that pneumococcal FtsE‐His is more soluble than its FtsE_Mtb_ homolog (Mir et al. 2006) and that FtsE_Spn_‐His binds ATP with an affinity comparable to other ABC transporter ATPase subunits. However, dimerization of purified pneumococcal FtsE‐His was not consistently detected, and at best, FtsE_Spn_‐His possesses a low ATPase activity alone or in the presence of purified FtsX‐His and PcsB‐His under the conditions tested so far.

**Table 2:** FtsE‐His exhibits a low ATPase activity under different reaction conditions^a^

Condition (molar ratio of proteins)	Reaction condition	ATPase specific activity (nmoles/min/mg)
FtsE‐His	Aqueous buffer	7.2 ± 2.9
FtsE‐His + FtsX‐His (1:1)	DDM detergent micelle	6.0 ± 2.1
FtsE‐His + FtsX‐His + PcsB‐His (1:1:2)	DDM detergent micelle	6.0 ± 1.5
FtsE‐His + FtsX‐His (1:1)	FtsX‐His nanodisc	8.7 ± 2.3
FtsE‐His + FtsX‐His + PcsB‐His (1:1:2)	FtsX‐His nanodisc	9.7 ± 3.8
MalFGK_2_ + MBP + maltose	Proteoliposome	1080 ± 7

a Proteins were purified and suspended in aqueous buffer, buffer containing 0.02% (wt/vol) DDM (n‐dodecyl‐*β*‐d‐maltoside), or nanodiscs as described in Materials and Methods, except for the maltose ABC transporter, which was reconstituted as described in (Orelle et al. 2008). ATPase specific activities were determined as described in Materials and Methods and (Orelle et al. 2008). Averages and standard deviations were from three independent determinations. Baseline (no protein) reading in aqueous buffer was 0.6 ± 0.2, which was subtracted from ATPase specific activities.

### Attempts to detect interactions of proteins in micelles and upon coexpression in *E. coli*

We made numerous attempts to detect binding between FtsX‐His and FtsE‐His in detergent micelles. These experiments were performed in the absence of ATP and in the presence of ATP and vanadate, which traps ADP at nucleotide binding sites by mimicking the transition state of the *γ*‐phosphate of ATP during hydrolysis (Sharma and Davidson 2000; Chen et al. 2001). Vanadate has been used extensively to trap transition states of ATPase subunits of ABC transporters (Chen et al. 2001). No peaks with higher molecular masses indicative of a complex between FtsX‐His and FtsE‐His were detected by analytical size‐exclusion chromatography (Fig. S5), and SDS‐PAGE showed that each respective peak contained only one protein and no detectable mixture of the two proteins (data not shown). Similarly, no complexes were detected for mixtures of FtsX‐His, FtsE‐His, and PcsB‐His in the above conditions (data not shown). Attempts to crosslink FtsX‐His and FtsE‐His in micelles with glutaraldehyde revealed an FtsX‐His dimer (see Fig. 4), but no additional complex containing FtsE‐His was detected (data not shown).

We also attempted to determine whether tagged FtsX and FtsE interact when overexpressed together in *E. coli*. The two proteins were expressed from replication‐compatible plasmids (Table S1), where one protein was His‐tagged and the other lacked an epitope tag. The purification of the His‐tagged proteins was performed as described for FtsX‐His through the affinity purification step, where cofactors were added at the step prior to membrane extraction (see Materials and Methods). Purification of membrane‐associated FtsE‐His did not pull down detectable coexpressed FtsX (data not shown). In the converse experiment, purification of membrane‐bound FtsX‐His was greatest when ADP or ATP and Mg^+2^ cofactors were added to extractions (data not shown). However, substantial amounts of untagged FtsE were recovered even under conditions of low FtsX‐His recovery, such as the absence of ADP or ATP and Mg^+2^, suggesting some form of nonspecific trapping or aggregation of FtsE. Therefore, attempts to detect complexes of purified FtsX‐His, FtsE‐His, and PcsB‐His or coexpressed tagged FtsX and FtsE have not yet been successful with the constructs and conditions tested so far.

### PcsB PG hydrolase interacts with charged phospholipids

**Figure 6:**
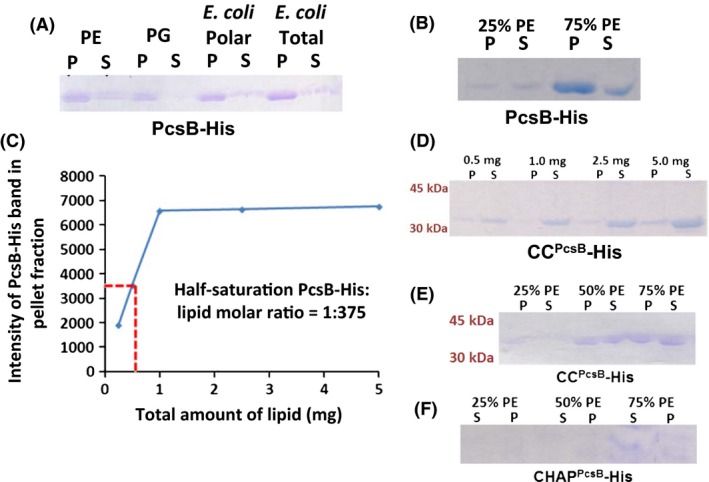
Liposome sedimentation binding assays of purified PcsB‐His and its CC^P^ ^csB^‐His and CHAP^P^ ^csB^‐His domains. Liposomes containing the indicated phospholipids (5 mg total lipids unless noted otherwise) were prepared, bound to PcsB‐His, CC^P^ ^csB^‐His, or CHAP^P^ ^csB^‐His, and collected by centrifugation as described in Materials and Methods. Amounts of protein bound to washed liposome pellets (P) or remaining in supernates (S) were determined by densitometry of SDS‐PAGE gels. (A) PcsB‐His binding to liposomes containing 50% phosphatidylcholine (neutral head group) + 50% phosphatidylethanolamine (positively charged head group) (PE); 50% phosphatidylcholine + 50% phosphatidylglycerol (negatively charged head group) (PG); *E. coli* polar lipids; or *E. coli* total lipids. (B) PcsB‐His binding to liposomes containing increasing proportions of PE (25% to 75%) compared to phosphatidylcholine (PC); (C) Binding assay of PcsB‐His to liposomes containing different total amounts of a mixture of 50% PE + 50% PC to determine the half‐saturation ratio of binding. Data points were averaged from two different experiments. (D) Binding of CC^P^ ^csB^‐His domain to liposomes containing different total amounts of a mixture of 50% PE + 50% PC. (E) CC^P^ ^csB^‐His binding to liposomes containing increasing proportions of PE (25–75%) compared to phosphatidylcholine (PC). (F) Minimal binding of CHAP^P^ ^csB^‐His to liposomes containing increasing proportions of PE (25–75%) compared to phosphatidylcholine (PC). All experiments were performed at least two times independently with similar results.

We purified a C‐terminal tagged version of the PcsB‐His PG hydrolase reported previously (Sham et al. 2011) and attempted to bind PcsB‐His to FtsX‐His nanodiscs. Although PcsB‐His appeared to bind to FtsX‐His nanodiscs, control experiments indicated that PcsB‐His also bound to empty nanodiscs lacking FtsX‐His (data not shown). This result suggested that PcsB‐His was likely binding to phospholipids in the nanodiscs. We used sedimentation of liposomes to assay for interactions between intact PcsB‐His and different phospholipids (see Materials and Methods). After addition of PcsB‐His, liposomes were collected by centrifugation, and the amount of PcsB‐His retained in washed liposome pellets was determined by SDS‐PAGE. PcsB‐His had a greater apparent affinity for liposomes containing phosphatidylethanolamine (PE) than for phosphatidylglycerol (PG), which has a positively or negatively charged head group, respectively (Fig. 6A and B). PcsB‐His also showed binding to *E. coli* polar and total lipids, which contain >50% PE and <25% PG. Increasing the percentage of PE compared to neutrally charged phosphatidylcholine (PC) in liposomes led to more PcsB‐His binding (Fig. 6B). Titration curves of PcsB‐His binding to liposomes containing increasing total amounts of a mixture of 50% PE + 50% PC indicated a molar half‐saturation ratio of one PcsB‐His to 380 phospholipid molecules (Fig. 6C).

PcsB contains two domains connected by a linker (Fig. 1) (Ng et al. 2003; Sham et al. 2011; Bartual et al. 2014). The coiled‐coil (CC^PcsB^‐His) and CHAP^PcsB^‐His domains were individually purified (see Materials and Methods) and used in liposome sedimentation assays. Similar to full‐length PcsB‐His, the CC^PcsB^‐His domain bound to liposomes containing positively charged PE, and more CC^PcsB^‐His bound to liposomes containing increasing amounts of PE compared to PC (Fig. 6D and E). By contrast, the CHAP^PcsB^‐His domain showed little detectable binding to liposomes containing PE (Fig. 6F) or PG (data not shown). Both the purified CC^PcsB^‐His and CHAP^PcsB^‐His domains showed affinities for mixtures of *E. coli* polar and total lipids (data not shown).

Some proteins change their subunit association in detergent micelles and phospholipid bilayers compared to aqueous solution (Bayburt et al. 2006; Borch and Hamann 2009; Lin et al. 2014; Chinthalapudi et al. 2015). In initial experiments, we examined the association state of PcsB‐His in aqueous solution compared to nonionic detergent micelles by size‐exclusion multiangle light‐scattering (SEC‐MALS) chromatography. In aqueous buffer (20 mmol/L Tris‐HCl, pH 8.0, 100 mmol/L NaCl), purified PcsB‐His at concentrations from 1.25 to 5.0 mg/mL was a monomer with a molecular mass of ≈41 kDa (data not shown), consistent with results reported previously showing that PcsB only dimerizes at relatively high protein concentrations (Bartual et al. 2014). However, in a micellar buffer, additionally containing nonionic 0.02% (wt/vol) DDM and 0.02% (wt/vol) C_12_E_8_ detergents (see Materials and Methods), PcsB‐His at 3.2 to 6.4 mg/mL eluted primarily as an oligomer, possibly a dimer, with a molecular mass of ≈106 kDa (data not shown). Together these initial results show that PcsB‐His binds to charged phospholipids, preferentially PE, likely through its CC domain, and PcsB‐His oligomerization may be influenced by interactions with hydrophobic nonionic detergents.

Finally, we examined whether detergent or PE phospholipid micellar mixtures containing purified FtsX‐His and FtsE‐His and Mg^+2^‐ATP or Mg^+2^‐ADP stimulated the PG hydrolase activity of PcsB‐His. Numerous unsuccessful attempts have been made to detect a PG hydrolase activity of intact purified PcsB, which is autoinhibited unless interacting with other proteins (Giefing et al. 2008; Sham et al. 2011; Bartual et al. 2014). Apparent PG hydrolase activity was observed for the purified CHAP^PcsB^ domain in zymograms, but this activity could not be recapitulated in solution (Bartual et al. 2014). Nevertheless, highly innovative domain‐swapping experiments of the CHAP domains of PcsB and LytF, which is a characterized PG hydrolase from another Streptococcus species, provide definitive evidence that PcsB functions as a PG hydrolase on pneumococcal cell surfaces (Bartual et al. 2014). We purified PG sacculi from wild‐type and PcsB depleted strains (Barendt et al. 2009; Boersma et al. 2015), and labeled the PG with fluorescamine as described in (Moser et al. 1988). PG hydrolase was assayed by the release of soluble fluorescent PG fragments from the insoluble PG preparations. The positive control containing purified pneumococcal LytA amidase exhibited PG hydrolase activity, whereas the combinations of PcsB‐His, FtsX‐His, FtsE‐His, and cofactors tested so far did not show significant release of fluorescent‐PG fragments compared to the no‐enzyme control (data not shown).

## Conclusions

This paper reports several steps on the path to reconstitute the FtsEX:PcsB complex biochemically. We worked out conditions to induce, stabilize, and purify the FtsX‐His membrane subunit in detergent micelles. We reconstituted FtsX‐His in phospholipid nanodiscs. We showed that FtsX‐His forms dimers in detergent micelles and likely in the nanodiscs. In addition, we worked out conditions to induce, stabilize, and purify the FtsE‐His ATPase subunit without the need for a renaturation step. We demonstrated that FtsE‐His binds an ATP analog with an affinity comparable to other ATPase subunits of ABC transporters and that FtsE‐His preparations have a low, but detectable, ATPase activity. In the course of working with nanodiscs, we found that PcsB‐His has an affinity for positively charged phospholipids, likely through interactions with the CC^PcsB^ domain.

However, specific binding complexes of purified FtsX‐His, FtsE‐His, and PcsB‐His in micelles have not yet been detected, and it will take considerably more preparative biochemical work to reconstitute a functioning FtsEX:PcsB complex in nanodiscs. One issue that needs to be resolved is the functionality of constructs of component proteins in bacterial cells. We attached several epitope tags, such as FLAG, to the C‐termini of PcsB and FtsE without deleterious effects on cell division or morphology (data not shown) (Sham et al. 2011), and the FtsE‐(C)‐His used in this study was active for binding of an ATP analog and exhibited low ATPase activity (Fig. 5; Table 2). In contrast, it has been much more challenging to identify tagged constructs to either terminus of FtsX that do not impair cellular function (data not shown). For this first characterization, we relied on His tags, which are robust for economical purification. His‐tagged versions of these proteins need to be characterized for function in cells, and conversely, His‐tag removal and other tags that do not disrupt cellular function need to be optimized in future reconstitution experiments. Moreover, it is likely that FtsE ATPase is substantially stimulated by interactions with other midcell division proteins, such as FtsZ (Fig. 1). However, little is known about these interactions at this point. Therefore, it will likely be necessary to add these additional proteins or to manipulate FtsE activity mutationally to stimulate FtsE ATPase in a purified system for kinetic studies and to rule out low‐level contaminating ATPase activity in FtsE preparations. Using these new protein constructs, reaction conditions including buffer and salt concentrations, pH, and temperature, will need to be optimized.

Another observation in this study has implications for the biochemical reconstitution of the FtsEX:PcsB complex and possibly for the assembly of this machine on pneumococcal cell surfaces. Phospholipid mixtures typically used for artificial membrane reconstitution in nanodiscs, such as soybean lipids (see Materials and Methods), contain positively charged phospholipids, like PE. The binding of PcsB‐His to positively charged phospholipids reported here may compete with binding to FtsX and prevent successful reconstitution of FtsEX:PcsB complexes. This raises the question of what happens during assembly on the pneumococcal cell surface. Notably, *S. pneumoniae* membranes are composed of a 50%:50% mixture of the negatively charged phospholipids, PG and cardiolipin, and do not contain any detectable positively charged phospholipids (Epand and Epand 2012). This phospholipid composition is in sharp contrast to that of well‐known gram‐negative bacteria, like *E. coli,* whose membranes contain ≈80% PE (Epand and Epand 2012). One implication of our results is that nanodiscs containing a 50%:50% mixture of PG and cardiolipin probably provide the best chance for reconstituting a functional FtsEX:PcsB complex.

**Figure 7:**
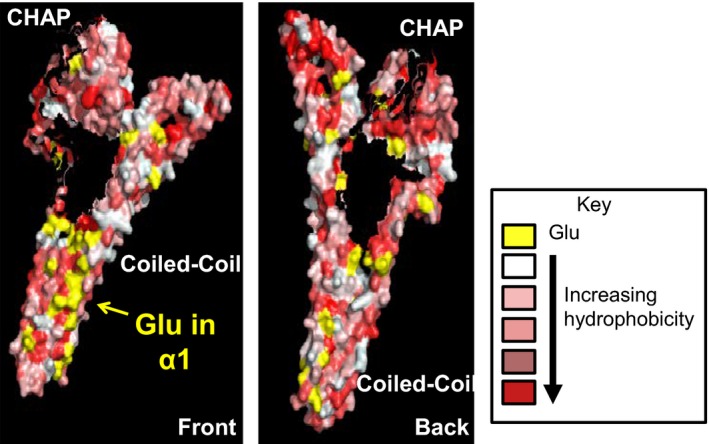
Hydrophobicity and distribution of negatively charged glutamates (yellow) on the surface of the structure of PcsB reported in (Bartual et al. 2014). The surface hydrophobicity was calculated as described in Materials and Methods. Two views are presented indicating the CHAP^P^ ^csB^ and CC^P^ ^csB^ domains and the abundance of Glu residues along one face of *α*‐helix 1 in the CC^P^ ^csB^ domain.

As a gram‐positive bacterium, *S. pneumoniae* lacks an outer membrane. PcsB has a processed signal sequence that is removed during export via the SecA‐SecYEG transporter (Barendt et al. 2009). Surprisingly, about 50% of total PcsB produced is secreted into the growth medium of cultures, and PcsB has been recognized as an immunodominant antigen in people infected with *S. pneumoniae* (Giefing et al. 2008; Sham et al. 2011). The affinity of PcsB and its CC^PcsB^ domain for positively charged phospholipids suggests that PcsB will be partially repelled by the highly negative membrane surface of *S. pneumoniae* cells. This repulsion may play a role in the substantial release of exported PcsB into the growth medium. The preference of PcsB‐His and CC^PcsB^‐His binding to positively charged phospholipids prompted us to examine the surface charge distribution on the structure of the “activated” conformation of the PcsB monomer reported in (Bartual et al. 2014). We noticed that glutamate residues are aligned along the whole length of alpha helix 1 (*α*1) in the CC^PcsB^ domain, providing an extended negatively charged patch (yellow, Fig. 7). This glutamate patch may lessen PcsB binding to the negatively charged membrane surface and could play some role in directing the CC^PcsB^ domain to bind to positively charged amino acids in ECL1^FtsX^ (Fig. 1) (Sham et al. 2011, 2013; Fu et al. 2015). The steps and timing of assembly of the FtsEX:PcsB complex may play some role in regulating PG cleavage at a specific time in the cell cycle and is an important topic for future study.

## Conflict of Interest

None declared.

## Supporting information


**Figure S1.** Western blot of FtsX‐GFP‐His with anti‐GFP antibody recovered in 1% (wt/vol) DDM‐extracted membrane supernates from strain IU6892 (BL21AI/pETCT *ftsX*‐ GFP‐His) induced with different inducers. Lane 1, no inducer; lane 2, 0.001% (wt/vol) arabinose; lane 3, 0.01% (wt/vol) arabinose; lane 4, 0.02% arabinose (wt/vol); lane 5 = 0.05% (wt/vol) arabinose; lane 6, 0.1% (wt/vol) arabinose; lane 7, 0.5 mmol/L IPTG; lane 8, 0.5 mmol/L IPTG + 0.01% (wt/vol) arabinose. Maximal induction was observed in lane 8, which is the condition that produced maximal fluorescence intensity in DDM‐extracted membrane supernates (see Table 1). The upper band is intact FtsX‐GFP‐His, whereas the lower bands present in all samples are degraded protein that retains the GFP epitope. Band intensities are not necessarily in the linear range of detection.
**Figure S2.** Growth curves of *E. coli* BL21DE3 cells expressing: no recombinant protein (blue diamond); FtsE‐His from plasmid pET22b‐*ftsE*‐His (strain IU4340) (red squares); FtsX‐His from plasmid pET22b‐*ftsX*‐His (strain IU6942) (green triangles); and FtsX‐His and FtsE from plasmids pET22b‐*ftsX*‐His and pACYC Duet‐*ftsE* (strain IU10589 (purple crosses). Strains are listed in Table S1. For growths, 50 mL of LB broth was inoculated with 50 *μ*L of overnight cultures of each strain and incubated with shaking at 25°C. At OD_600_ = 0.5, expression of recombinant proteins was induced with 1 mmol/L IPTG (time = 0), and OD_600_ was monitored with time. Data points were averages of three independent growths. Growth of strains expressing recombinant proteins slowed down significantly after induction, but the cultures did not lyse while expressing recombinant proteins.
**Figure S3.** Fluorescence‐detection size‐exclusion chromatography (FSEC) to optimize the concentration of (A) detergents and (B) glycerol for FtsX‐GFP‐His purification. Besides the indicated detergents and glycerol, elution buffer contained 50 mmol/L Tris‐HCl pH 8.0, 200 mmol/L NaCl, and columns were run at 0.5 mL per min. A total of 15% (vol/vol) glycerol was added to the buffers in panel A. See Materials and Methods for additional details.
**Figure S4.** Western blot showing that FtsE‐His runs at 30 kDa, instead of at 26 kDa on SDS‐PAGE. FtsE‐His was overexpressed in strain IU4340 (BL21DE3/pET22b‐*ftsE*‐His) that was induced for 20 h at 16°C following addition of 1 mmol/L IPTG. FtsE‐His was purified as described in Materials and Methods, and purified FtsE‐His was analyzed by SDS‐PAGE with Coomassie blue staining (left panel). Purified MBP‐His was included as a positive control for detecting His‐tagged proteins. The positions of standards from a size standard ladder are indicated. The gel was then western blotted as described in Materials and Methods (right panel). The Coomassie‐stained gel shows that FtsE‐His preparations contain at least two faint contaminant bands, and the western blot show that the prominent 30 kDa band corresponds to FtsE‐His.
**Figure S5.** Association studies of purified FtsE‐His and FtsX‐His proteins using analytical size‐exclusion chromatography (Superdex 200 30/100 column in 50 mmol/L Tris‐ HCl pH 8.0, 200 mmol/L NaCl, 5% (vol/vol) glycerol, 0.02% (wt/vol) DDM, 0.02% (wt vol) C12E8), performed as described in Materials and Methods. A. FtsE‐His + FtsX‐His (1:1 molar ratio) B. FtsE‐His + FtsX‐His (1:1 molar ratio) + 5 mmol/L ATP + 5 mmol/L MgCl_2_ + 0.5 mmol/L vanadate to allow trapping. FtsX‐His alone (green, lines); FtsE‐His alone (blue lines); mixtures of FtsE‐His + FtsX‐His (red lines). See text for additional details.
**Table S1.** Bacterial strains and primers used in this study.
**Table S2.** Detergent screening for optimal extraction of FtsX‐GFP‐His from membranes.
**Table S3**. Optimization of FtsX‐GFP‐His expression by varying IPTG concentrations.

## References

[mbo3366-bib-0001] Alvarez, F. J. , C. Orelle , and A. L. Davidson . 2010 Functional reconstitution of an ABC transporter in nanodiscs for use in electron paramagnetic resonance spectroscopy. J. Am. Chem. Soc. 132:9513–9515. 20578693 10.1021/ja104047c PMC2927203

[mbo3366-bib-0002] Arends, S. J. , R. J. Kustusch , and D. S. Weiss . 2009 ATP‐binding site lesions in FtsE impair cell division. J. Bacteriol. 191:3772–3784. 19376877 10.1128/JB.00179-09 PMC2698383

[mbo3366-bib-0003] Barendt, S. M. , A. D. Land , L. T. Sham , W. L. Ng , H. C. Tsui , R. J. Arnold , et al. 2009 Influences of capsule on cell shape and chain formation of wild‐type and *pcsB* mutants of serotype 2 *Streptococcus pneumoniae* . J. Bacteriol. 191:3024–3040. 19270090 10.1128/JB.01505-08 PMC2681789

[mbo3366-bib-0004] Barendt, S. M. , L. T. Sham , and M. E. Winkler . 2011 Characterization of mutants deficient in the L, D‐carboxypeptidase (DacB) and WalRK (VicRK) regulon, involved in peptidoglycan maturation of Streptococcus pneumoniae serotype 2 strain D39. J. Bacteriol. 193:2290–2300. 21378199 10.1128/JB.01555-10 PMC3133071

[mbo3366-bib-0005] Bartual, S. G. , D. Straume , G. A. Stamsas , I. G. Munoz , C. Alfonso , M. Martinez‐Ripoll , et al. 2014 Structural basis of PcsB‐mediated cell separation in *Streptococcus pneumoniae* . Nat. Commun. 5:3842. doi: 10.1038/ncomms4842. 24804636 10.1038/ncomms4842

[mbo3366-bib-0006] Bayburt, T. H. , Y. V. Grinkova , and S. G. Sligar . 2006 Assembly of single bacteriorhodopsin trimers in bilayer nanodiscs. Arch. Biochem. Biophys. 450:215–222. 16620766 10.1016/j.abb.2006.03.013

[mbo3366-bib-0007] Boersma, M. J. , E. Kuru , J. T. Rittichier , M. S. Vannieuwenhze , Y. V. Brun , and M. E. Winkler . 2015 Minimal peptidoglycan (PG) turnover in wild‐type and PG hydrolase and cell division mutants of *Streptococcus pneumoniae* D39 growing planktonically and in host‐relevant biofilms. J. Bacteriol. 197:3472–3485. 26303829 10.1128/JB.00541-15 PMC4621067

[mbo3366-bib-0008] Borch, J. , and T. Hamann . 2009 The nanodisc: a novel tool for membrane protein studies. Biol. Chem. 390:805–814. 19453280 10.1515/BC.2009.091

[mbo3366-bib-0009] Chen, J. , S. Sharma , F. A. Quiocho , and A. L. Davidson . 2001 Trapping the transition state of an ATP‐binding cassette transporter: evidence for a concerted mechanism of maltose transport. Proc. Natl Acad. Sci. USA 98:1525–1530. 11171984 10.1073/pnas.041542498 PMC29290

[mbo3366-bib-0010] Chinthalapudi, K. , D. N. Patil , E. S. Rangarajan , C. Rader , and T. Izard . 2015 Lipid‐directed vinculin dimerization. Biochemistry 54:2758–2768. 25880222 10.1021/acs.biochem.5b00015

[mbo3366-bib-0011] Davidson, A. L. , E. Dassa , C. Orelle , and J. Chen 2008 Structure, function, and evolution of bacterial ATP‐binding cassette systems. Microbiol. Mol. Biol. Rev. 72:317–364, table of contents. 18535149 10.1128/MMBR.00031-07 PMC2415747

[mbo3366-bib-0012] Denisov, I. G. , Y. V. Grinkova , A. A. Lazarides , and S. G. Sligar . 2004 Directed self‐assembly of monodisperse phospholipid bilayer nanodiscs with controlled size. J. Am. Chem. Soc. 24:3477–3487. 10.1021/ja0393574 15025475

[mbo3366-bib-0013] Egan, A. J. , and W. Vollmer . 2013 The physiology of bacterial cell division. Ann. N. Y. Acad. Sci. 1277:8–28. 23215820 10.1111/j.1749-6632.2012.06818.x

[mbo3366-bib-0014] Egan, A. J. , J. Biboy , I. Van't Veer , E. Breukink , and W. Vollmer . 2015 Activities and regulation of peptidoglycan synthases. Philos. Trans. R. Soc. Lond. B Biol. Sci., 370:1679doi:10.1098/rstb. 10.1098/rstb.2015.0031 PMC4632607 26370943

[mbo3366-bib-0015] Eisenberg, D. , E. Schwarz , M. Komaromy , and R. Wall . 1984 Analysis of membrane and surface protein sequences with the hydrophobic moment plot. J. Mol. Biol. 179:125–142. 6502707 10.1016/0022-2836(84)90309-7

[mbo3366-bib-0016] Epand, R. M. , and R. F. Epand . 2012 Functional consequences of the lateral organization of biological membranes Chapter 7. Pp. 133–152 *in* Yeagle Philip. L., ed. The structure of biological membranes. CRC Press, Boca Raton, FL.

[mbo3366-bib-0017] Foster, J. E. , Q. Sheng , J. R. McClain , M. Bures , T. I. Nicas , K. Henry , et al. 2004 Kinetic and mechanistic analyses of new classes of inhibitors of two‐component signal transduction systems using a coupled assay containing HpkA‐DrrA from *Thermotoga maritima* . Microbiology 150:885–896. 15073298 10.1099/mic.0.26824-0

[mbo3366-bib-0018] Fu, Y. , K. E. Bruce , B. Rued , M. E. Winkler , and D. P. Giedroc . 2015 H, C, N resonance assignments of the extracellular loop 1 domain (ECL1) of *Streptococcus pneumoniae* D39 FtsX, an essential cell division protein. Biomol NMR Assign 10:89–92. 26370567 10.1007/s12104-015-9644-9 PMC4789122

[mbo3366-bib-0019] Gasteiger, E. , A. Gattiker , C. Hoogland , I. Ivanyi , R. D. Appel , and A. Bairoch 2003 ExPASy: The proteimics server for in‐depth protein knowledge and analysis Nuc Acid Res. 31:3784–3788. 10.1093/nar/gkg563 PMC168970 12824418

[mbo3366-bib-0020] Giefing, C. , A. L. Meinke , M. Hanner , T. Henics , M. D. Bui , D. Gelbmann , et al. 2008 Discovery of a novel class of highly conserved vaccine antigens using genomic scale antigenic fingerprinting of pneumococcus with human antibodies. J. Exp. Med. 205:117–131. 18166586 10.1084/jem.20071168 PMC2234372

[mbo3366-bib-0021] Giefing‐Kroll, C. , K. E. Jelencsics , S. Reipert , and E. Nagy . 2011 Absence of pneumococcal PcsB is associated with overexpression of LysM domain‐containing proteins. Microbiology 157:1897–1909. 21474534 10.1099/mic.0.045211-0

[mbo3366-bib-0022] Kawate, T. , and E. Gouaux . 2006 Fluorescence‐detection size‐exclusion chromatography for precrystallization screening of integral membrane proteins. Structure 14:673–681. 16615909 10.1016/j.str.2006.01.013

[mbo3366-bib-0023] de Leeuw, E. , B. Graham , G. J. Phillips , C. M. Ten Hagen‐Jongman , B. Oudega , and J. Luirink . 1999 Molecular characterization of *Escherichia coli* FtsE and FtsX. Mol. Microbiol. 31:983–993. 10048040 10.1046/j.1365-2958.1999.01245.x

[mbo3366-bib-0024] Lin, W. C. , L. Iversen , H. L. Tu , C. Rhodes , S. M. Christensen , J. S. Iwig , et al. 2014 H‐Ras forms dimers on membrane surfaces via a protein‐protein interface. Proc. Natl Acad. Sci. USA 111:2996–3001. 24516166 10.1073/pnas.1321155111 PMC3939930

[mbo3366-bib-0025] Massidda, O. , L. Novakova , and W. Vollmer . 2013 From models to pathogens: how much have we learned about *Streptococcus pneumoniae* cell division? Environ. Microbiol. 15:3133–3157. 23848140 10.1111/1462-2920.12189

[mbo3366-bib-0026] Mavrici, D. , M. J. Marakalala , J. M. Holton , D. M. Prigozhin , C. L. Gee , Y. J. Zhang , et al. 2014 *Mycobacterium tuberculosis* FtsX extracellular domain activates the peptidoglycan hydrolase, RipC. Proc. Natl Acad. Sci. USA 111:8037–8042. 24843173 10.1073/pnas.1321812111 PMC4050617

[mbo3366-bib-0027] Meisner, J. , P. Montero Llopis , L. T. Sham , E. Garner , T. G. Bernhardt , and D. Z. Rudner . 2013 FtsEX is required for CwlO peptidoglycan hydrolase activity during cell wall elongation in *Bacillus subtilis* . Mol. Microbiol. 89:1069–1083. 23855774 10.1111/mmi.12330 PMC3786131

[mbo3366-bib-0028] Mesnage, S. , F. Chau , L. Dubost , and M. Arthur . 2008 Role of N‐acetylglucosaminidase and N‐acetylmuramidase activities in *Enterococcus faecalis* peptidoglycan metabolism. J. Biol. Chem. 283:19845–19853. 18490448 10.1074/jbc.M802323200

[mbo3366-bib-0029] Mir, M. A. , H. S. Rajeswari , U. Veeraraghavan , and P. Ajitkumar . 2006 Molecular characterization of ABC transporter type FtsE and FtsX proteins of *Mycobacterium tuberculosis* . Arch. Microbiol. 185:147–158. 16416128 10.1007/s00203-005-0079-z

[mbo3366-bib-0030] Mir, M. A. , M. Arumugam , S. Mondal , H. S. Rajeswari , S. Ramakumar , and P. Ajitkumar . 2015 *Mycobacterium tuberculosis* cell division protein, FtsE, is an ATPase in dimeric form. Protein J. 34:35–47. 25511207 10.1007/s10930-014-9593-7

[mbo3366-bib-0031] Moser, I. , F. Pittner , and P. Dworsky . 1988 A rapid fluorimetric test for lysozyme with purified fluorescamine‐labelled peptidoglycan. J. Biochem. Biophys. Methods 17:249–252. 3149658 10.1016/0165-022x(88)90048-6

[mbo3366-bib-0032] Narayanan, A. , M. Ridilla , and D. A. Yernool . 2011 Restrained expression, a method to overproduce toxic membrane proteins by exploiting operator‐repressor interactions. Protein Sci. 20:51–61. 21031485 10.1002/pro.535 PMC3047061

[mbo3366-bib-0033] Nath, A. , W. M. Atkins , and S. G. Sligar . 2007 Applications of phospholipid bilayer nanodiscs in the study of membranes and membrane proteins. Biochemistry 46:2059–2069. 17263563 10.1021/bi602371n

[mbo3366-bib-0034] Ng, W. L. , G. T. Robertson , K. M. Kazmierczak , J. Zhao , R. Gilmour , and M. E. Winkler . 2003 Constitutive expression of PcsB suppresses the requirement for the essential VicR (YycF) response regulator in *Streptococcus pneumoniae* R6. Mol. Microbiol. 50:1647–1663. 14651645 10.1046/j.1365-2958.2003.03806.x

[mbo3366-bib-0035] Ng, W. L. , K. M. Kazmierczak , and M. E. Winkler . 2004 Defective cell wall synthesis in *Streptococcus pneumoniae* R6 depleted for the essential PcsB putative murein hydrolase or the VicR (YycF) response regulator. Mol. Microbiol. 53:1161–1175. 15306019 10.1111/j.1365-2958.2004.04196.x

[mbo3366-bib-0036] Orelle, C. , T. Ayvaz , R. M. Everly , C. S. Klug , and A. L. Davidson . 2008 Both maltose‐binding protein and ATP are required for nucleotide‐binding domain closure in the intact maltose ABC transporter. Proc. Natl Acad. Sci. USA 105:12837–12842. 18725638 10.1073/pnas.0803799105 PMC2529024

[mbo3366-bib-0037] Peters, N. T. , C. Morlot , D. C. Yang , T. Uehara , T. Vernet , and T. G. Bernhardt . 2013 Structure‐function analysis of the LytM domain of EnvC, an activator of cell wall remodelling at the *Escherichia coli* division site. Mol. Microbiol. 89:690–701. 23796240 10.1111/mmi.12304 PMC3923381

[mbo3366-bib-0038] Potluri, L. , A. Karczmarek , J. Verheul , A. Piette , J. M. Wilkin , N. Werth , et al. 2010 Septal and lateral wall localization of PBP5, the major D, D‐carboxypeptidase of Escherichia coli, requires substrate recognition and membrane attachment. Mol. Microbiol. 77:300–323. 20545860 10.1111/j.1365-2958.2010.07205.x PMC2909392

[mbo3366-bib-0039] Rees, D. C. , E. Johnson , and O. Lewinson . 2009 ABC transporters; the power to change. Nat. Rev. Molec. Cell Biol. 10:218–227. 19234479 10.1038/nrm2646 PMC2830722

[mbo3366-bib-0040] Schmidt, K. L. , N. D. Peterson , R. J. Kustusch , M. C. Wissel , B. Graham , G. J. Phillips , et al. 2004 A predicted ABC transporter, FtsEX, is needed for cell division in *Escherichia coli* . J. Bacteriol. 186:785–793. 14729705 10.1128/JB.186.3.785-793.2004 PMC321481

[mbo3366-bib-0041] Sham, L. T. , S. M. Barendt , K. E. Kopecky , and M. E. Winkler . 2011 Essential PcsB putative peptidoglycan hydrolase interacts with the essential FtsX_*Spn*_ cell division protein in *Streptococcus pneumoniae* D39. Proc. Natl Acad. Sci. USA 108:E1061–E1069. 22006325 10.1073/pnas.1108323108 PMC3215045

[mbo3366-bib-0042] Sham, L. T. , K. R. Jensen , K. E. Bruce , and M. E. Winkler . 2013 Involvement of FtsE ATPase and FtsX extracellular loops 1 and 2 in FtsEX‐PcsB complex function in cell division of *Streptococcus pneumoniae* D39. MBio, 4: pii: e00431‐13. doi: 10.1128/mBio.00431‐13. 23860769 10.1128/mBio.00431-13 PMC3735124

[mbo3366-bib-0043] Sharma, S. , and A. L. Davidson . 2000 Vanadate‐induced trapping of nucleotides by purified maltose transport complex requires ATP hydrolysis. J. Bacteriol. 182:6570–6576. 11073897 10.1128/jb.182.23.6570-6576.2000 PMC111395

[mbo3366-bib-0044] Stewart, R. C. , R. Vanbruggen , D. D. Ellefson , and A. J. Wolfe . 1998 TNP‐ATP and TNP‐ADP as probes of the nucleotide binding site of CheA, the histidine protein kinase in the chemotaxis signal transduction pathway of *Escherichia coli* . Biochemistry 37:12269–12279. 9724541 10.1021/bi980970n

[mbo3366-bib-0045] Typas, A. , M. Banzhaf , C. A. Gross , and W. Vollmer . 2012 From the regulation of peptidoglycan synthesis to bacterial growth and morphology. Nat. Rev. Microbiol. 10:123–136. 10.1038/nrmicro2677 PMC5433867 22203377

[mbo3366-bib-0046] Vollmer, W. 2012 Bacterial growth does require peptidoglycan hydrolases. Mol. Microbiol. 86:1031–1035. 23066944 10.1111/mmi.12059

[mbo3366-bib-0047] Vollmer, W. , B. Joris , P. Charlier , and S. Foster . 2008 Bacterial peptidoglycan (murein) hydrolases. FEMS Microbiol. Rev. 32:259–286. 18266855 10.1111/j.1574-6976.2007.00099.x

[mbo3366-bib-0048] Weiss, D. S. 2004 Bacterial cell division and the septal ring. Mol. Microbiol. 54:588–597. 15491352 10.1111/j.1365-2958.2004.04283.x

[mbo3366-bib-0049] Yang, D. C. , N. T. Peters , K. R. Parzych , T. Uehara , M. Markovski , and T. G. Bernhardt . 2011 An ATP‐binding cassette transporter‐like complex governs cell‐wall hydrolysis at the bacterial cytokinetic ring. Proc. Natl Acad. Sci. USA 108:E1052–E1060. 22006326 10.1073/pnas.1107780108 PMC3215046

[mbo3366-bib-0050] Yang, D. C. , K. Tan , A. Joachimiak , and T. G. Bernhardt . 2012 A conformational switch controls cell wall‐remodelling enzymes required for bacterial cell division. Mol. Microbiol. 85:768–781. 22715947 10.1111/j.1365-2958.2012.08138.x PMC3418388

[mbo3366-bib-0051] Young, K. D. 2010 Bacterial shape: two‐dimensional questions and possibilities. Annu. Rev. Microbiol. 64:223–240. 20825347 10.1146/annurev.micro.112408.134102 PMC3559087

