# Supplementary material for: Biochemical characterization of essential cell division proteins FtsX and FtsE that mediate peptidoglycan hydrolysis by PcsB in Streptococcus pneumoniae
Source: Microbiologyopen. 2016 May 10;5(5):738–52. doi: 10.1002/mbo3.366 (PMC5061712; doi:10.1002/mbo3.366)
Supplement: Supplementary file 1 — Figure S1. Western blot of FtsX‐GFP‐His with anti‐GFP antibody recovered in 1% (wt/vol) DDM‐extracted membrane supernates from strain IU6892 (BL21AI/pETCT ftsX‐ GFP‐His) induced with different inducers. Lane 1, no inducer; lane 2, 0.001% (wt/vol) arabinose; lane 3, 0.01% (wt/vol) arabinose; lane 4, 0.02% arabinose (wt/vol); lane 5 = 0.05% (wt/vol) arabinose; lane 6, 0.1% (wt/vol) arabinose; lane 7, 0.5 mmol/L IPTG; lane 8, 0.5 mmol/L IPTG + 0.01% (wt/vol) arabinose. Maximal induction was observed in lane 8, which is the condition that produced maximal fluorescence intensity in DDM‐extracted membrane supernates (see Table 1). The upper band is intact FtsX‐GFP‐His, whereas the lower bands present in all samples are degraded protein that retains the GFP epitope. Band intensities are not necessarily in the linear range of detection. Figure S2. Growth curves of E. coli BL21DE3 cells expressing: no recombinant protein (blue diamond); FtsE‐His from plasmid pET22b‐ftsE‐His (strain IU4340) (red squares); FtsX‐His from plasmid pET22b‐ftsX‐His (strain IU6942) (green triangles); and FtsX‐His and FtsE from plasmids pET22b‐ftsX‐His and pACYC Duet‐ftsE (strain IU10589 (purple crosses). Strains are listed in Table S1. For growths, 50 mL of LB broth was inoculated with 50 μL of overnight cultures of each strain and incubated with shaking at 25°C. At OD600 = 0.5, expression of recombinant proteins was induced with 1 mmol/L IPTG (time = 0), and OD600 was monitored with time. Data points were averages of three independent growths. Growth of strains expressing recombinant proteins slowed down significantly after induction, but the cultures did not lyse while expressing recombinant proteins. Figure S3. Fluorescence‐detection size‐exclusion chromatography (FSEC) to optimize the concentration of (A) detergents and (B) glycerol for FtsX‐GFP‐His purification. Besides the indicated detergents and glycerol, elution buffer contained 50 mmol/L Tris‐HCl pH 8.0, 200 mmol/L NaCl, and columns were r [file MBO3-5-738-s001.pdf]

SUPPLEMENTAL INFORMATION

Biochemical Characterization of Essential Cell Division Proteins FtsX and FtsE That Mediate Peptidoglycan Hydrolysis by PcsB in *Streptococcus pneumoniae*

Ruchika Bajaj[1], Kevin E. Bruce[2], Amy L. Davidson[1‡], Britta E. Rued[2], Cynthia V. Stauffacher[1], and Malcolm E. Winkler*[2]

[1]Department of Biological Sciences, Purdue University, West Lafayette, IN 47907 and [2]Department of Biology, Indiana University Bloomington, Bloomington, IN 47405, USA

Running title: Reconstitution of Pneumococcal FtsX in Nanodiscs

Supplemental Tables: S1-S3

Supplemental Figure Legends: S1-S5

Corresponding author:

Malcolm E. Winkler

Department of Biology

Indiana University Bloomington

1001 E. 3rd St.

Bloomington, IN 47405 USA

Phone: 812-856-1318

E-mail: winklerm@indiana.edu

Table S1. Bacterial strains and primers used in this study

Strain number	*E. coli* strain	Plasmid[a]	Antibiotic resistance marker[b]	Construction[c]	Description[a]	Reference
IU6892	BL21AI	pETCT-*ftsX*-GFP-His$_8$	Kan	*ftsX* cloned into pET-CT-GFP-HIS-LIC with *Nde*I and *Xho*I	C-terminal GFP and His$_8$ tagged FtsX	This study
Primer		Sequence (5'-3'; RE sites in **bold**)[d]				
KB103		CTTTAAGAAGGAGATATA**CATATG**TCATTGCCATTGAAAATGGCCGTG				
KB100		CAGAAAATATAAATTTTC**CTCGAG**AATCTTCAAGAATCGGCGCATGGA				
IU6884	BL21AI	pEVL4-His$_6$-GFP-*ftsX*	Kan	*ftsX* cloned into pEV-L4 with *Kpn*I and *Eco*RI	N-terminal GFP and His$_6$ tagged FtsX	This study
Primer		Sequence (5'-3'; RE sites in **bold**)				
KB104		GATGAACTCTACCGA**GGTACC**GAGAACCTGTACTTCCAATCCTCATTGCCATT				
KB102		ACGTGCCAC**GAATTC**CTAAATCTTCAAGAATCGGCG				
IU10693	BL21DE3	pETCT-*ftsX*-GFP-His$_8$	Kan	See IU6892 above	C-terminal GFP and His$_8$ tagged FtsX	This study
IU6942	BL21DE3	pET22b-*ftsX*-His$_6$	Amp	*ftsX* cloned into pET22b with *Nde*I and *Not*I	C-terminal His$_6$ tagged FtsX	This study
Primer		Sequence (5'-3'; RE sites in **bold**)				
KB119		CGGTAG**CATATG**TCATTGCCATTGAAAATGGCCGTG				
KB120		AGT**GCGGCCGC**AATCTTCAAGAATCGGCGCATGG				
IU4340	BL21DE3	pET22b-*ftsE*-His$_6$	Amp	*ftsE* cloned into pET22b with *Nde*I and *Not*I	C-terminal His$_6$ tagged FtsE	This study
Primer		Sequence (5'-3'; RE sites in **bold**)				
CS132		ATA**CATATG**TCAATTATTGAAATGAGAGATGTCGTT				
CS133		AGT**GCGGCCGC**ATCATCGTATCCATACTCTCCTTTTGATTC				
IU6917	BL21DE3	pACYC-Duet-*ftsE*	Chl	*ftsE* cloned into pACYC Duet with *Nde*I and *Xho*I	Untagged FtsE	This study
Primer		Sequence (5'-3'; RE sites in **bold**)				
KB96		CGGTAG**CATATG**TCAATTATTGAAATGAGAGATGTC				
KB118		CCG**CTCGAG**CTAATCATCGTATCCATACTCTCC				
IU6454	BL21DE3	pACYC-Duet-*ftsX*	Chl	*ftsX* cloned into pACYC Duet with *Nde*I and *Kpn*I	Untagged FtsX	This study

Primer	Sequence (5’-3’; RE sites in **bold**)					
CS241	CCGG**CATATG**ATTAGTAGATTTTTTCGCCATTTATTTG					
CS242	CCGG**GGTACC**AATCTTCAAGAATCGGCGCAT					
IU10588	BL21DE3	pET22b-*ftsE*-His$_6$ pACYC-Duet-*ftsX*	Amp, Chl	*ftsE* cloned into pET22b with *Nde*I and *Not*I *ftsX* cloned in pACYC Duet with *Nde*I and *Kpn*I	Co-expressed C-terminal His$_6$ tagged FtsE and untagged FtsX	This study
Primer	Sequence (5’-3’; RE sites in **bold**)					
CS132	ATA**CATATG**TCAATTATTGAAATGAGAGATGTCGTT					
CS133	AGT**GCGGCCGC**ATCATCGTATCCATACTCTCCTTTTGATTC					
CS241	CCGG**CATATG**ATTAGTAGATTTTTTCGCCATTTATTTG					
CS242	CCGG**GGTACC**AATCTTCAAGAATCGGCGCAT					
IU10589	BL21DE3	pET22b-*ftsX*-His$_6$ pACYC-Duet-*ftsE*	Amp, Chl	*ftsX* cloned into pET22b with *Nde*I and *Not*I *ftsE* cloned into pACYC Duet with *Nde*I and *Xho*I	Co-expressed C-terminal His$_6$ tagged FtsX and untagged FtsE	This study
Primer	Sequence (5’-3’; RE sites in **bold**)					
KB119	CGGTAG**CATATG**TCATTGCCATTGAAAATGGCCGTG					
KB120	AGT**GCGGCCGC**AATCTTCAAGAATCGGCGCATGG					
KB96	CGGTAG**CATATG**TCAATTATTGAAATGAGAGATGTC					
KB118	CCG**CTCGAG**CTAATCATCGTATCCATACTCTCC					
IU1614	BL21DE3	pET22b-*pcsB*$^{\Delta N27}$-His$_6$	Amp, Chl	*pcsB*$^{\Delta N27}$ cloned into pET22b with *Nde*I and *Not*I	C-terminal His$_6$ tagged PcsB with first 27 residues deleted	Barendt *et al*., 2009
Primer	Sequence (5’-3’; RE sites in **bold**)					
WN119	CTCGAG**GCGGCCGC**ATCTGCATAAATATATGTAACAAAACC					
WN117	**CATATG**GAAACGACTGATGACAAAATTGCTG					
IU4561	BL21AI	pET22b-*pcsB*$^{28\text{-}259}$ (CC)-His$_6$	Amp	*pcsB*$^{28\text{-}259}$ (CC) cloned into pET22b with *Nde*I and *Not*I	C-terminal His$_6$ tagged PcsB CC domain	Sham *et al*., 2011
Primer	Sequence (5’-3’; RE sites in **bold**)					
WN117	**CATATG**GAAACGACTGATGACAAAATTGCTG					
KG002	ACGGAATA**GCGGCCGC**AGCTGTTAAGTTAGTGTTTGCTGAAGCA					
IU1791	BL21DE3	pET22b-*pcsB*$^{271\text{-}392}$ (CHAP)-	Amp, Chl	*pcsB*$^{271\text{-}392}$ (CHAP) cloned into pET22b	C-terminal His$_6$ tagged PcsB CHAP	Sham *et al*., 2011

		His_6		with *Nde*I and *Not*I	domain	
Primer		Sequence (5’-3’; RE sites in **bold**)				
SC001		GGAATTC**CATATG**GTCCGTGCAAAAGTTCGTCCAACAT				
WN119		CTCGAG**GCGGCCGC**ATCTGCATAAATATATGTAACAAAACC				

[a]His_8 and His_6 tags are abbreviated as “His” in the text.

[b]Antibiotic resistance markers: Amp, ampicillin; Kan, kanamycin; Chl, chloramphenicol.

[c]See Materials and Methods for additional information about plasmid constructs.

[d]RE site, restriction enzyme cleavage site built into primers to allow cloning of amplicons synthesized by high-fidelity PCR.

Table S2. Detergent screening for optimal extraction of FtsX-GFP-His from membranes[a]

Detergent[b]	Fluorescence intensity recovered in detergent-extracted membrane supernates (RFU)[c]
None	899
1% DDM<br>2% DDM	**2,902**<br>**3,231**
1% OG<br>2% OG	2,441<br>2,038
1% Fos12<br>2% Fos12	2,539<br>3,470
1% Sodium Cholate<br>2% Sodium Cholate	2,671<br>984
1% LDAO<br>2% LDAO	**3,539**<br>**3,051**
1% Anergent<br>2% Anergent	**3,412**<br>**2,742**
1% CHAPS<br>2% CHAPS	2,181<br>1,718

[a]Strain IU6892 (BL21AI/pETCT-*ftsX*-GFP-His) was grown and induced as described in Materials and Methods.

[b]FtsX-GFP-His was extracted from membranes with the concentrations of detergents shown as described in Materials and Methods. Abbreviations: DDM = n-dodecyl-β-D-maltoside; OG = n-octyl-β-D-glucoside; Fos12 = foscholine-12 = n-dodecylphosphocholine; LDAO = n-dodecyl-N, N-dimethylamine oxide, CHAPS = [3-[(3-cholamidopropyl)-dimethylammonio]-1-propane sulfonate] Detergents were obtained from Anatrace, Inc.

[c]RFU = random fluorescence unit. Most effective detergents for FtsX-GFP-His solubilization from membranes are emboldened. DDM was the most effective and economical detergent and was incorporated in the purification procedures in Materials and Methods.

Table S3. Optimization of FtsX-GFP-His expression by varying IPTG concentrations[a]

[IPTG] (mM)	Fluorescence intensity (RFU)[b]		
	Cell lysates	Membrane resuspension	DDM-extracted membrane supernate[c]
0	434	140	115
0.1	7,240	7,640	5,503
0.5	10,936	12,651	11,646
**1.0**	**8,074**	**13,216**	**13,816**

[a]Strain IU10693 (BL21DE3/pETCT-*ftsX*-GFP-His) was grown and induced as described in Materials and Methods.

[b]RFU = random fluorescence unit.

[c]FtsX-GFP-His was extracted from membranes with 1% (wt/vol) DDM as described in Materials and Methods.

SUPPLEMENTAL FIGURE LEGENDS

Fig. S1. Western blot of FtsX-GFP-His with anti-GFP antibody recovered in 1% (wt/vol) DDM-extracted membrane supernates from strain IU6892 (BL21AI/ pETCT *ftsX*-GFP-His) induced with different inducers. Lane 1, no inducer; lane 2, 0.001% (wt/vol) arabinose; lane 3, 0.01% (wt/vol) arabinose; lane 4, 0.02% arabinose (wt/vol); lane 5= 0.05% (wt/vol) arabinose; lane 6, 0.1% (wt/vol) arabinose; lane 7, 0.5 mM IPTG; lane 8, 0.5mM IPTG + 0.01% (wt/vol) arabinose. Maximal induction was observed in lane 8, which is the condition that produced maximal fluorescence intensity in DDM-extracted membrane supernates (see Table 1). The upper band is intact FtsX-GFP-His, whereas the lower bands present in all samples are degraded protein that retains the GFP epitope. Band intensities are not necessarily in the linear range of detection.

Fig S2. Growth curves of *E. coli* BL21DE3 cells expressing: no recombinant protein (blue diamond); FtsE-His from plasmid pET22b-*ftsE*-His (strain IU4340) (red squares); FtsX-His from plasmid pET22b-*ftsX*-His (strain IU6942) (green triangles); and FtsX-His and FtsE from plasmids pET22b-*ftsX*-His and pACYC Duet-*ftsE* (strain IU10589 (purple crosses). Strains are listed in Table S1. For growths, 50 mL of LB broth was inoculated with 50 μL of overnight cultures of each strain and incubated with shaking at 25°C. At OD_{600} = 0.5, recombinant proteins were induced with 1 mM IPTG (time = 0), and OD_{600} was monitored with time. Data points are averages of three independent growths. Growth of strains expressing recombinant proteins slowed down significantly after induction, but the cultures did not lyse while expressed recombinant proteins.

Fig. S3. Fluorescence-detection size-exclusion chromatography (FSEC) to optimize the concentration of (A) detergents and (B) glycerol for FtsX-GFP-His purification. Besides the indicated detergents and glycerol, elution buffer contained 50 mM Tris-HCl pH 8.0, 200 mM NaCl, and columns were run at 0.5 mL per min. 15% (vol/vol) glycerol was added to the buffers in panel A. See Materials and Methods for additional details.

Fig S4. Western blot showing that FtsE-His runs at 30 kDa, instead of at 26 kDa on SDS-PAGE. FtsE-His was overexpressed in strain IU4340 (BL21DE3/pET22b-*ftsE*-His) that was induced for 20 h at 16°C following addition of 1 mM IPTG. FtsE-His was purified as described in Materials and Methods, and purified FtsE-His was analyzed by SDS-PAGE with Coomassie blue staining (left panel). Purified MBP-His was included as a positive control for detecting His-tagged proteins. The positions of standards from a size-standard ladder are indicated. The gel was then Western blotted as described in Materials and Methods (right panel). The Coomassie-stained gel shows that FtsE-His preparations contain at least two faint contaminant bands, and the Western blot show that the prominent 30 kDa band corresponds to FtsE-His.

Fig. S5. Association studies of purified FtsE-His and FtsX-His proteins using analytical size-exclusion chromatography (Superdex 200 30/100 column in 50 mM Tris-HCl pH 8.0, 200 mM NaCl, 5% (vol/vol) glycerol, 0.02% (wt/vol) DDM, 0.02% (wt vol) $C_{12}E_8$), performed as described in Materials and Methods. A. FtsE-His + FtsX-His (1:1 molar ratio) B. FtsE-His + FtsX-His (1:1 molar ratio) + 5 mM ATP + 5 mM $MgCl_2$ + 0.5 mM vanadate to allow trapping. FtsX-His alone (green, lines); FtsE-His alone (blue lines); mixtures of FtsE-His + FtsX-His (red lines). See text for additional details.

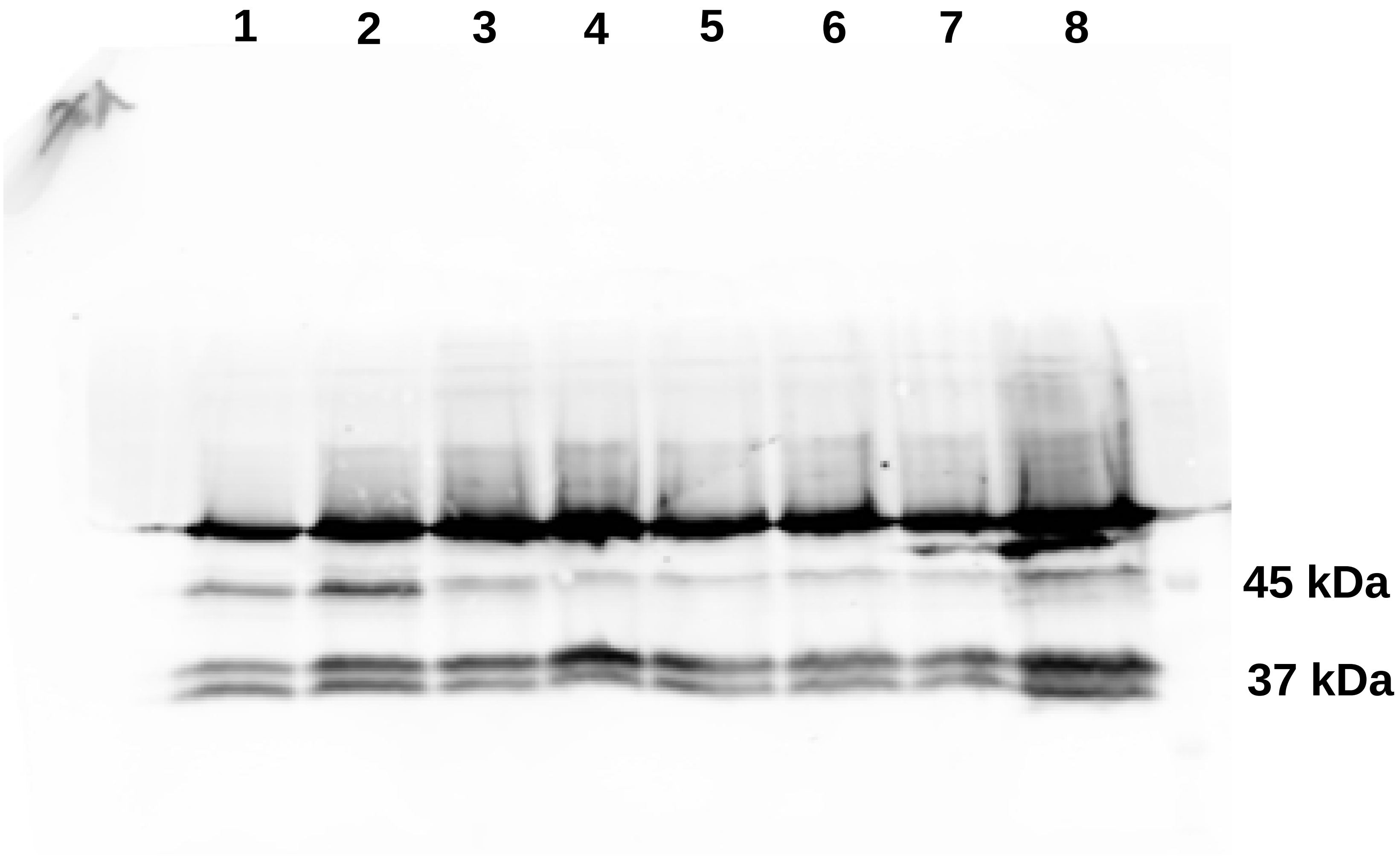

Fig. S1

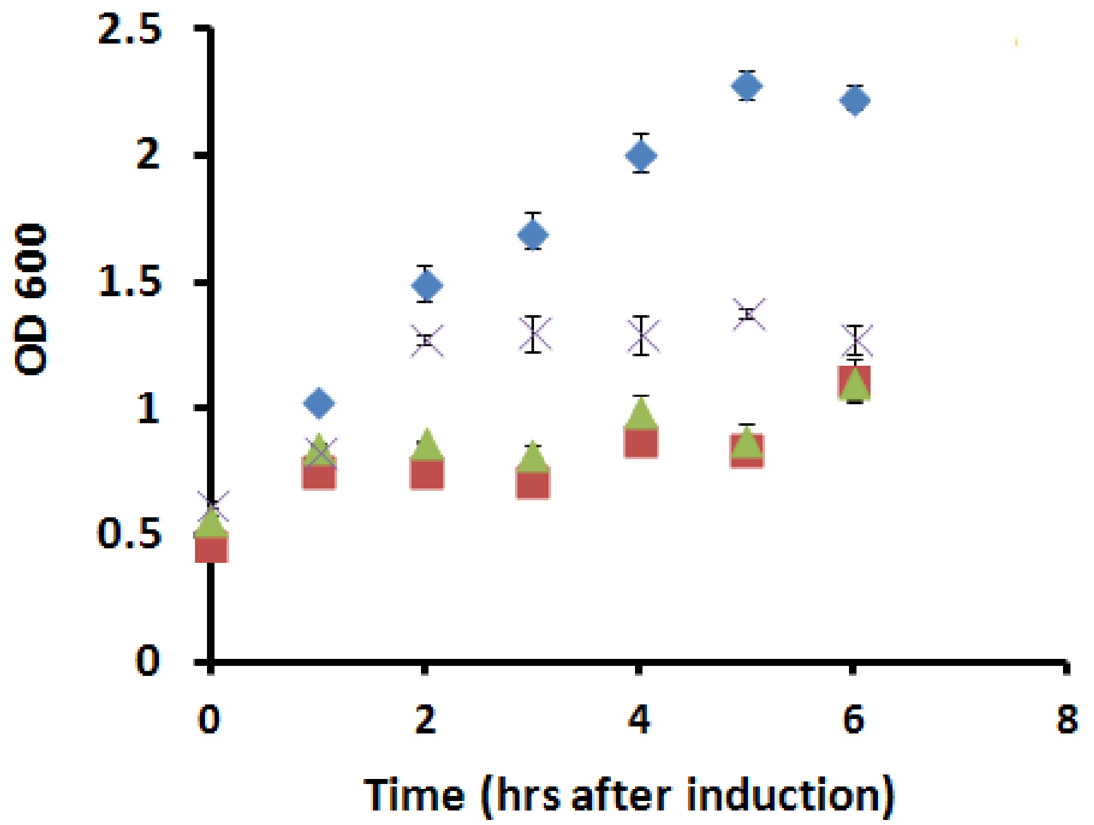

Fig. S2

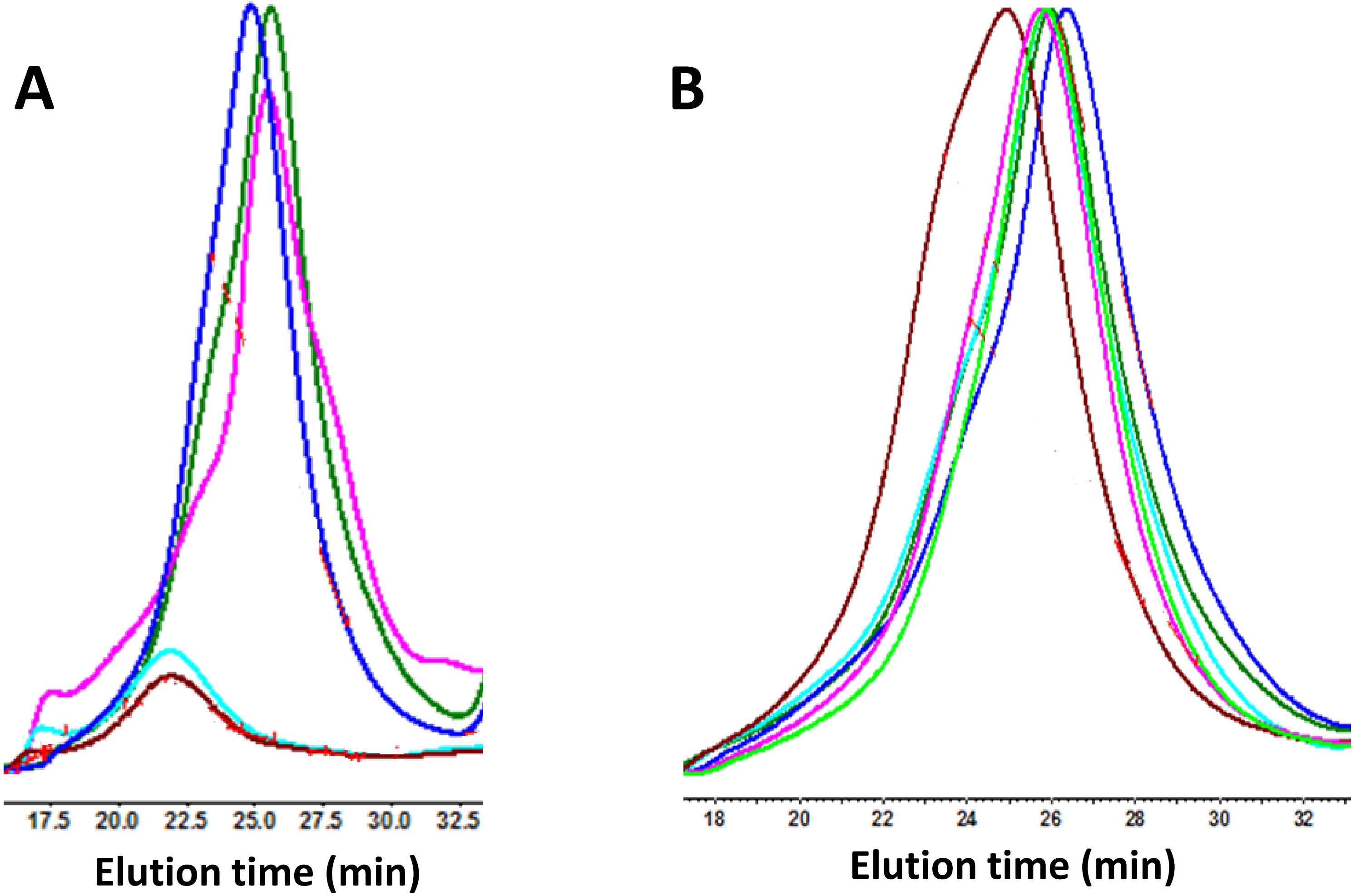

0.02% DDM(—),
0.05% DDM(—),
0.02% C12E8(—),
0.05% C12E8(—),
0.02% DDM + 0.02% C12E8(—)

0% glycerol, 0.02% DDM (—),
5% glycerol, 0.02% DDM (—),
10% glycerol, 0.02% DDM (—),
0% glycerol, 0.02% DDM + 0.02% C12E8 (—),
5% glycerol, 0.02% DDM + 0.02% C12E8 (—),
10% glycerol, 0.02% DDM + 0.02% C12E8 (—).

Fig. S3

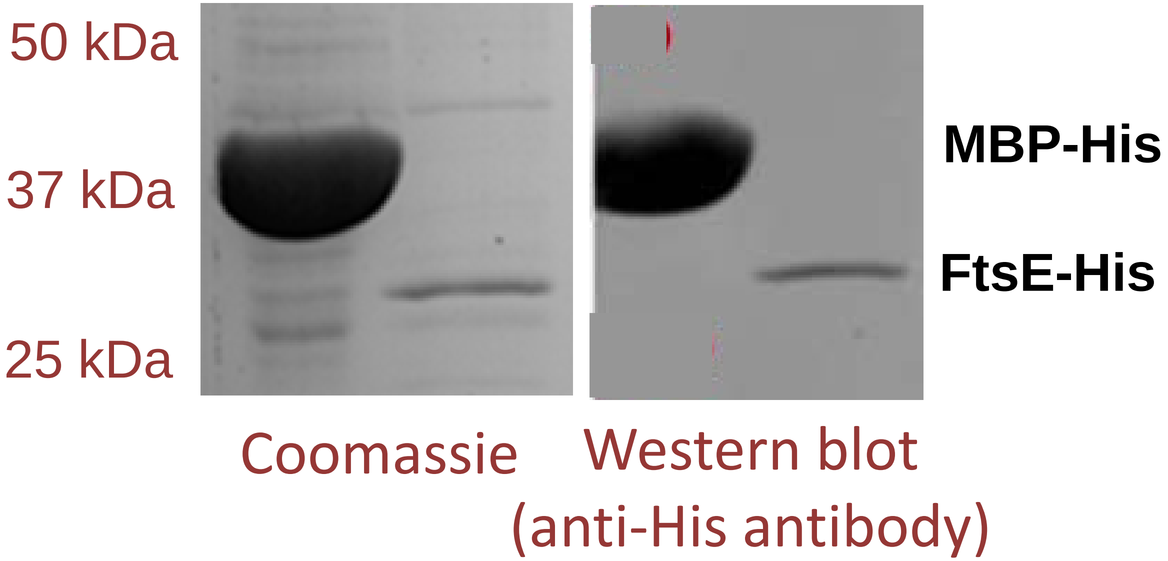
50 kDa
37 kDa
25 kDa
MBP-His
FtsE-His
Coomassie
Western blot
(anti-His antibody)

Fig. S4

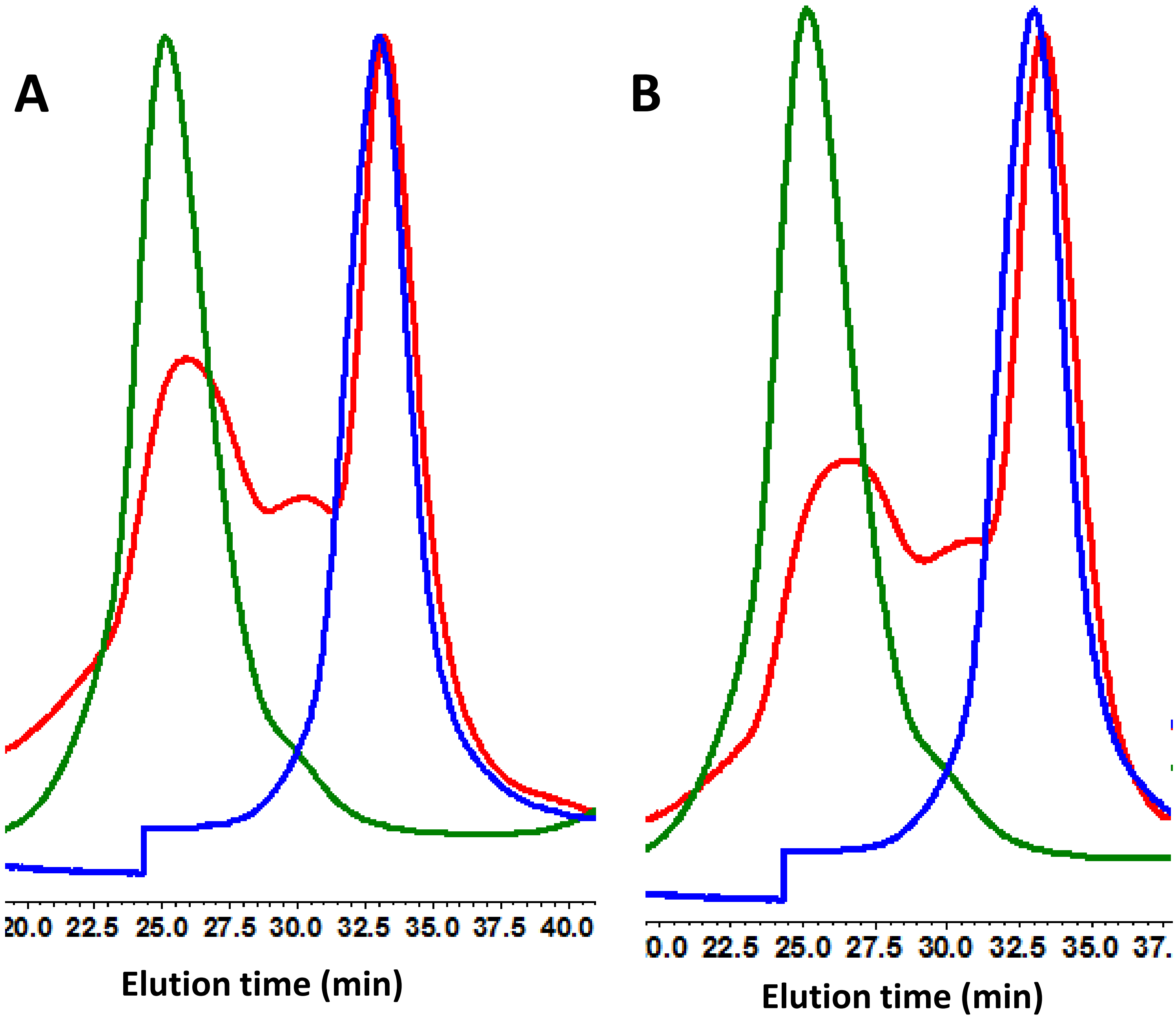
A
B
20.0 22.5 25.0 27.5 30.0 32.5 35.0 37.5 40.0
Elution time (min)
0.0 22.5 25.0 27.5 30.0 32.5 35.0 37.
Elution time (min)

Fig. S5
